# Fission yeast Bgs1 glucan synthase participates in the control of growth polarity and membrane traffic

**DOI:** 10.1016/j.isci.2024.110477

**Published:** 2024-07-08

**Authors:** Mariona Ramos, Rebeca Martín-García, M. Ángeles Curto, Laura Gómez-Delgado, M. Belén Moreno, Mamiko Sato, Elvira Portales, Masako Osumi, Sergio A. Rincón, Pilar Pérez, Juan C. Ribas, Juan C.G. Cortés

**Affiliations:** 1 Instituto de Biología Funcional y Genómica, Consejo Superior de Investigaciones Científicas (CSIC) and Universidad de Salamanca, Salamanca, Spain; 2 Laboratory of Electron Microscopy and Bio-imaging Center, Japan Women’s University, 2-8-1 Mejirodai, Bunkyo-ku, Tokyo, Japan; 3 Integrated Imaging Research Support (IIRS), Villa Royal Hirakawa 103, 1-7-5 Hirakawa-cho, Chiyoda-ku, Tokyo, Japan

**Keywords:** Functional aspects of cell biology, Mycology, Organizational aspects of cell biology

## Abstract

Rod-shaped fission yeast grows through cell wall expansion at poles and septum, synthesized by essential glucan synthases. Bgs1 synthesizes the linear β(1,3)glucan of primary septum at cytokinesis. Linear β(1,3)glucan is also present in the wall poles, suggesting additional Bgs1 roles in growth polarity. Our study reveals an essential collaboration between Bgs1 and Tea1-Tea4, but not other polarity factors, in controlling growth polarity. Simultaneous absence of Bgs1 function and Tea1-Tea4 causes complete loss of growth polarity, spread of other glucan synthases, and spherical cell formation, indicating this defect is specifically due to linear β(1,3)glucan absence. Furthermore, linear β(1,3)glucan absence induces actin patches delocalization and sterols spread, which are ultimately responsible for the growth polarity loss without Tea1-Tea4. This suggests strong similarities in Bgs1 functions controlling actin structures during cytokinesis and polarized growth. Collectively, our findings unveil that cell wall β(1,3)glucan regulates polarized growth, like the equivalent extracellular matrix in neuronal cells.

## Highlights

• Bgs1 β-glucan cooperates with the Tea1-Tea4 complex in the control of growth polarity
• Bgs1 β-glucan regulates the polarized localization of actin patches and sterols
• Actin patches collaborate with Tea1-Tea4 in the regulation of growth polarity
• Bgs1 glucan synthase is necessary for proper endocytosis and membrane traffic

## Graphical abstract

**Figure:**
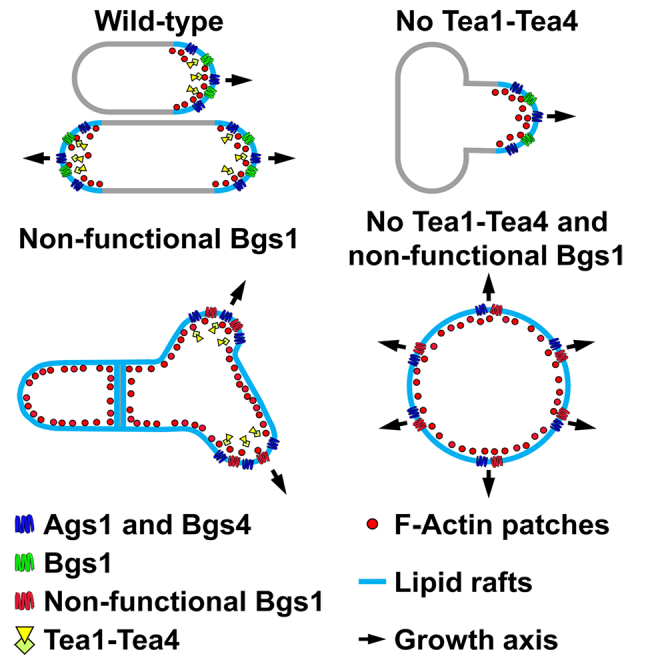

## Introduction

Cell polarity is crucial for cell function and organ development. Loss of cellular polarity is a common hallmark in cancer progression and dissemination.^1^ The fission yeast *Schizosaccharomyces pombe* exhibits highly polarized cells with a distinctive rod-shaped morphology, achieved by limiting growth to the cell tips. As in other eukaryotes, the GTPase Cdc42 is a major regulator of cell polarity and dimensions.^2^^,^^3^ However, the specific mechanisms governing the establishment and restriction of growth to the cell tips remain incompletely understood.

Sterol-rich membrane (SRM) domains delineate the sites of growth in fission yeast and other fungal species.^4^^,^^5^^,^^6^ These SRMs are initially randomly distributed before growth initiation, providing platforms for the assembly of polarity factors and proteins required for growth.^7^ The microtubule cytoskeleton polarizes and maintains SRM domains at the tip by delivering the signaling complex composed of the kelch domain-containing protein Tea1 and the adaptor protein Tea4.^7^^,^^8^^,^^9^^,^^10^ The Tea1-Tea4 complex binds to the microtubule plus end by interaction with the end-binding protein Mal3 and the kinesin Tea2.^11^ Upon reaching the tip cortex, the Tea1-Tea4 complex signals to proteins involved in positioning polarized growth through formation of actin cables in a complex termed polarisome, including Bud6 and the formin For3, which is activated by Cdc42.^8^^,^^12^^,^^13^ Furthermore, the Tea1-Tea4 complex signals to the DYRK-family kinase Pom1^14^ and the protein phosphatase 1 (PP1) catalytic subunit Dis2.^15^ Pom1 plays a crucial role in coordinating polarized growth and cell cycle progression.^16^^,^^17^ Cells lacking Pom1 exhibit defects in both cell polarity and cell size at mitosis, along with misplaced division septa.^14^ Tea4 binds to Dis2, which dephosphorylates Pom1 to maintain its localization at the membrane of the cell tip.^18^

In fungal cells, the deposition of the cell wall follows the establishment of growth polarity, and, reciprocally, the composition and mechanical properties of the cell wall influence the shape and growth of walled cells.^19^^,^^20^ In the fission yeast cell wall, the major polysaccharides are branched β(1,3)glucan and α(1,3)glucan, synthesized by the essential glucan synthases Bgs4 and Ags1/Mok1, respectively. Bgs4 and Ags1 are involved in the maintenance of cell integrity. Consequently, repression of *bgs4*^+^ or *ags1*^*+*^ results in cell lysis, and mutants of Bgs4 and Ags1 present a weak cell wall and rounded morphology that is suppressed by osmotic stabilization.^21^^,^^22^^,^^23^^,^^24^^,^^25^ Extracellular branched β(1,3)glucan is required for cytokinesis, connecting cell wall to plasma membrane and contractile ring, and regulating essential intracellular functions of cytokinesis.^25^ An analogous role has been suggested for the extracellular matrix, the functional equivalent of the cell wall in animal cells.^26^^,^^27^ Linear β(1,3)glucan is a minor polymer that forms the primary septum during cytokinesis and is also present in minor amounts at the cell wall in the growing ends. Its presence decreases in the lateral walls as the cell lengthens, likely due to cell wall remodeling and maturation.^28^^,^^29^ The essential β(1,3)glucan synthase Bgs1 is responsible for the synthesis of this minor linear β(1,3)glucan, but is not involved in cell integrity.^28^^,^^30^^,^^31^ Accordingly, repression of *bgs1*^+^ results in branched and multiseptated cells with aberrant primary septa that cannot be suppressed by osmotic stabilization, highlighting the significance of Bgs1 in cell wall dynamics, but not in cell integrity.^28^^,^^32^ During cytokinesis, all synthases are detected in the cell middle, where Bgs1 and Bgs4 confer stability to the actomyosin ring structure.^25^^,^^30^^,^^32^^,^^33^ Notably, Bgs1 precedes other synthases to the cleavage furrow, playing a crucial role in the focalized location of other glucan synthases,^31^^,^^33^ and its cooperation with paxillin Pxl1, calcineurin phosphatase, F-BAR protein Cdc15, and Sbg1 is essential for actomyosin contractile ring stability and septum formation.^32^^,^^34^^,^^35^^,^^36^ Similarly, after cytokinesis Bgs1 is the first glucan synthase to arrive to the cell pole and resume polarized growth, probably regulating other glucan synthases as it does in cytokinesis.^21^^,^^22^^,^^37^

Our study unveils that in addition to synthesizing linear β(1,3)glucan during cytokinesis, Bgs1 and its extracellular linear β(1,3)glucan play an essential role in maintaining polarized growth in cooperation with the Tea1-Tea4 complex. Simultaneous absence of Bgs1 β(1,3)glucan and Tea1-Tea4 complex causes the loss of growth polarity and formation of spherical cells, suggesting a redundant safety mechanism to control growth polarity. We demonstrate that (i) Bgs1 activity is crucial for the polarized localization of actin patches and formation of SRM domains, and (ii) this delocalization of actin patches when Bgs1 activity fails is the ultimately responsible for the loss of growth polarity in the absence of the Tea1-Tea4 complex. This shows the high similarities in the essential functions of Bgs1 controlling the stability of actin structures, actomyosin ring during cytokinesis and actin patches during polarized growth. Furthermore, Bgs1 is also necessary for the proper processes of endocytosis, membrane traffic, and endosome and vacuole formation. This study provides valuable insights between the function of cell wall β(1,3)glucan in the control of growth polarity and an analogous role of the equivalent extracellular matrix in animal cells,^38^^,^^39^^,^^40^ as it has been shown for cytokinesis, reinforcing the strong functional similarities between both structures.

## Results

### Bgs1 cooperates specifically with the Tea1-Tea4 complex in the maintenance of growth polarity

**Figure 1:**
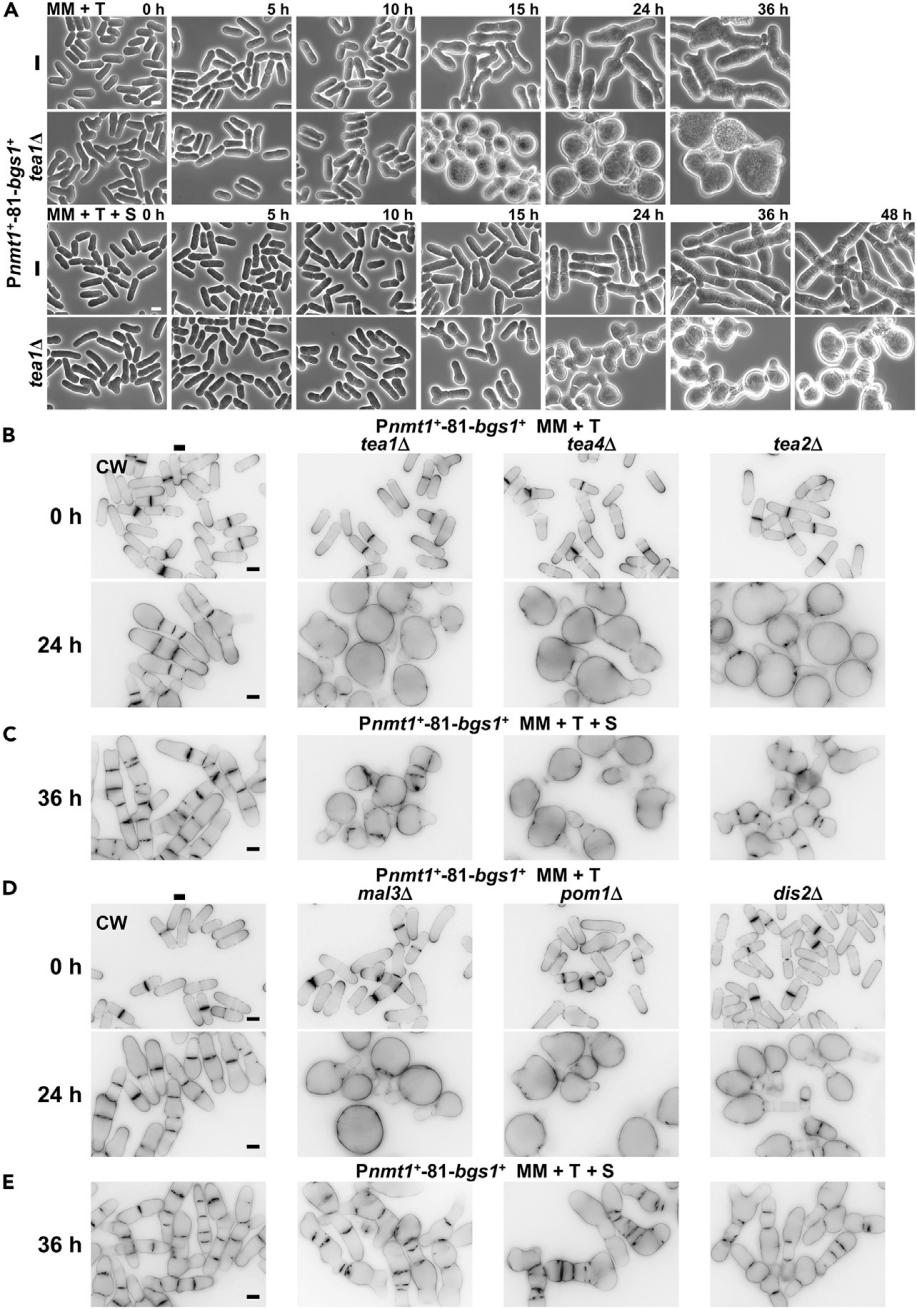
Bgs1 cooperates with the Tea1-Tea4 complex and Pom1 kinase in the essential control of growth polarity (A) Time course phase contrast micrographs of control and *tea1*Δ cells carrying *bgs1*^+^ under the control of the thiamine-repressible *nmt1*^+^*-*81 promoter (P*nmt1*^*+*^-81-*bgs1*^+^). Cells were grown to early log-phase at 28°C in minimal medium (MM) without thiamine (*bgs1*^+^ ON, − T) and without (upper panels) or with 1.3 M sorbitol (+S, lower panels). The cells were then transferred to MM with thiamine (*bgs1*^+^ OFF, + T) either in the absence (MM + T, upper panels) or presence of sorbitol (MM + T + S, lower panels) and imaged for phase contrast at the indicated times with thiamine (0, 5, 10, 15, 24, and 36 h for MM + T, and the same times plus 48 h for MM + T + S). (B and C) Fluorescence micrographs of calcofluor white (CW)-stained control, *tea1*Δ, *tea4*Δ, and *tea2*Δ cells carrying *bgs1*^+^ under the control of the *nmt1*^*+*^-81 promoter. Cells were grown to early log-phase at 28°C in MM (B) or MM + S (C). Then, the cells were transferred to MM + T (B) or MM + T + S (C) and imaged for CW fluorescence at the indicated times with thiamine (0 and 24 h for MM + T, and 36 h for MM + T + S). (D and E) Control, *mal3*Δ, *pom1*Δ, and *dis2*Δ cells carrying P*nmt1*^*+*^-81-*bgs1*^+^ were grown to early log-phase at 28°C in MM (D) or MM + S (E), transferred to MM + T (D) or MM + T + S (E), and imaged as in B and C at the indicated times with thiamine (0 and 24 h for MM + T, and 36 h for MM + T + S). Scale bars, 5 μm. See also Figure S1 and Table S1.

Fission yeast Bgs1 is an essential protein that localizes to the septum edge during cytokinesis and is responsible for the synthesis of linear β(1,3)glucan of the primary septum.^30^ To analyze the effect of Bgs1 absence, a time course repression of the *bgs1*^+^ gene was performed by adding thiamine to cells carrying *bgs1*^+^ under the control of the *nmt1*^+^*-*81 promoter, which is a very-low expression version of the thiamine-repressible *nmt1*^+^ promoter.^41^ Cell growth progressively slows down during *bgs1*^+^ repression and therefore, for time course analysis standard repression times separated by a single cell cycle interval were selected as reported.^28^ After 24 h of *bgs1*^+^ repression, the cells appeared elongated and multiseptated, both in the absence and presence of the osmotic stabilizer sorbitol. This phenotype continued to worsen with longer repression periods (Figure 1A), consistent with previous findings.^28^^,^^32^ Shorter periods of *bgs1*^+^ repression resulted in a milder phenotype of elongated cells with one septum after 15 h without sorbitol. No apparent phenotype was observed with sorbitol under these conditions (Figure 1A). It is worth noting that the expression level of Bgs1 reportedly decreases by 300-fold after 10 h of *bgs1*^+^ repression, and continues decreasing to undetectable levels after 15 h, at which point no apparent phenotype is still detectable. This indicates that the Bgs1 level required for synthesizing normal septa and maintaining a wild-type (WT) phenotype is extremely low. The multiseptated phenotype is only produced when the Bgs1 amount is undetectable.^28^ Therefore, for morphological analysis of Bgs1 absence, standard repression times of 15 and 24 h without sorbitol, and 24 and 36 h with sorbitol, were selected, aligning with previous studies.^28^^,^^32^^,^^33^

During the time course, Bgs1-depleted cells occasionally exhibited some ectopic cell growth defects, including bifurcated tips with two areas of cell growth (Figure S1A, arrow), suggesting a role of Bgs1 in growth polarity. This observation is supported by the presence of both Bgs1 and its linear β(1,3)glucan at the cell tips during interphase.^28^^,^^37^ It has been shown that Bgs1 cooperates with paxillin Pxl1, calcineurin, F-BAR protein Cdc15, and Sbg1 for contractile ring stability and septum formation during cytokinesis.^32^^,^^34^^,^^35^^,^^36^ However, nothing is known about the role of Bgs1 in polarized growth and the possible existence of a similar cooperation with polarity factors. To investigate the potential role of Bgs1 in polarized growth, we analyzed through a time course study whether there is an additive effect between the absence of Bgs1 and the absence of the polarity establishment proteins Tea1 or Tea4.^8^^,^^9^^,^^10^ Surprisingly, instead of the typical branched and multiseptated cells maintaining growth polarity even after 72 h of *bgs1*^+^ repression (100% elongated and multiseptated cells; Figure S1A), depletion of Bgs1 in the absence of Tea1 or Tea4 led to the emergence of rounded cells after 15 h in the absence or 24 h in the presence of sorbitol, forming large spherical cells after 24 or 36 h, respectively (100% rounded cells; Figures 1A, 1B, and 1C; Table 1). Septation was affected in the spherical cells, mainly in the absence of sorbitol, some with incomplete and the majority without septa. In addition, the emergence of this rounded phenotype was always coincident with the emergence of elongated and multiseptated phenotype when Tea1 and Tea4 are present, both in the absence and presence of sorbitol (Figures 1A, 1B, and 1C). As a control (0 h of repression), cells only lacking Tea1 or Tea4 displayed rod or T-shaped phenotypes (100% elongated cells; Figures 1A and 1B). These results suggest that (i) Bgs1 collaborates with the polarity markers Tea1 and Tea4 in a process necessary for maintaining polarized growth in fission yeast, and (ii) a minor cell wall synthesis defect that may produce the sole Bgs1 absence is not the cause of the rounded morphology.

**Table 1:** Summary of the morphological phenotypes exhibited by *bgs1*^*+*^ repression in the absence of genes involved in cell polarity

Strain	Cell morphology with thiamine (+T)	
	Minimal Medium (MM + T)	MM plus sorbitol (MM + S + T)
P*nmt1*-81-*bgs1*^*+*^	Elongated and multiseptated	Elongated and multiseptated
*tea1*Δ P*nmt1*-81-*bgs1*^*+*^	Rounded	Rounded
*tea4*Δ P*nmt1*-81-*bgs1*^*+*^	Rounded	Rounded
*tea2*Δ P*nmt1*-81-*bgs1*^*+*^	Rounded	Elongated and multiseptated
*mal3*Δ P*nmt1*-81-*bgs1*^*+*^	Rounded	Elongated and multiseptated
*pom1*Δ P*nmt1*-81-*bgs1*^*+*^	Rounded	Elongated and multiseptated
*dis2*Δ P*nmt1*-81-*bgs1*^*+*^	Rounded	Elongated and multiseptated
*mod5*Δ P*nmt1*-81-*bgs1*^*+*^	Elongated and multiseptated	Elongated and multiseptated
*bud6*Δ P*nmt1*-81-*bgs1*^*+*^	Elongated and multiseptated	Elongated and multiseptated
*for3*Δ P*nmt1*-81-*bgs1*^*+*^	Elongated and multiseptated	Elongated and multiseptated
*tea3*Δ P*nmt1*-81-*bgs1*^*+*^	Elongated and multiseptated	Elongated and multiseptated
*cdc42-D76G* P*nmt1*-81-*bgs1*^*+*^	Elongated and multiseptated	Elongated and multiseptated
*cdc42-L160S* P*nmt1*-81-*bgs1*^*+*^	Elongated and multiseptated	Elongated and multiseptated
*shk1-34* (*orb2-34*) P*nmt1*-81-*bgs1*^*+*^	Elongated and multiseptated	Elongated and multiseptated
*shk2Δ* P*nmt1*-81-*bgs1*^*+*^	Elongated and multiseptated	Elongated and multiseptated

See also Figure S1 and Table S1.

Tea2 and Mal3 are functionally related to Tea1 and Tea4, playing crucial roles in the microtubule-mediated transport of the polarity markers to the poles.^42^^,^^43^ Depletion of Bgs1 in cells lacking either Tea2 or Mal3 in the absence of sorbitol induced the same spherical phenotype observed in cells lacking both Bgs1 and Tea1 or Tea4 (Figures 1B and 1D; Table 1). However, Bgs1-depleted *tea2*Δ cells grown with sorbitol acquired a multiseptated “string of pearls” morphology of chained rounded cells, displaying more septa than cells generated during Bgs1 depletion in the absence of Tea1 or Tea4 (Figure 1C). Similarly, Bgs1-depleted *mal3*Δ cells grown with sorbitol did not induce a rounded morphology, displaying the multiseptated phenotype of Bgs1-depleted cells (Figure 1E; Table 1). The DYRK-family kinase Pom1 acts downstream of the Tea1-Tea4 complex^14^; and Tea4 also recruits Dis2, one of the PP1 catalytic subunits,^15^ promoting dephosphorylation of Pom1 and allowing the binding of this kinase to the plasma membrane at the cell ends.^18^ Bgs1 depletion in cells lacking Pom1 or Dis2 induced the loss of growth polarity in the absence of sorbitol (Figure 1D; Table 1). In the presence of sorbitol, these cells maintained polarized growth and formed multiple septa, similar to *tea2*Δ or *mal3*Δ cells lacking Bgs1 (Figure 1E; Table 1). The mild Tea2, Mal3, Pom1, and Dis2 rounded phenotype in the absence of Bgs1, and only detected in the absence of sorbitol, suggests the presence of either residual Tea1-Tea4 or residual activity downstream of Pom1. In addition, since the defective septation phenotype promoted by Bgs1 absence was more severe in the absence of the Tea1-Tea4 complex than in cells lacking Tea2, Mal3, Pom1, or Dis2, it is possible that Tea1 and Tea4 have additional functions in septation, which would be independent of both microtubules and Pom1 functions. Globally, all these results suggest that Bgs1 cooperates with the Tea1-Tea4 complex, and likely with Pom1, in the definition of the growing poles.

### The actin cables are not involved in the cooperation between Bgs1 and the Tea1-Tea4 complex in the regulation of polarized growth

We also examined the effect of *bgs1*^+^ repression in the absence of Mod5, a prenylated protein involved in the anchorage of Tea1 to the plasma membrane at the cell tip.^44^ However, our results showed that the absence of Mod5 did not alter the cell morphology during Bgs1 depletion even in the absence of sorbitol (Figure S1B; Table 1). Once in the membrane, the Tea1-Tea4 complex recruits proteins of the polarisome, like the formin For3 and its associated partners, including the nucleation-promoting factor Bud6.^45^ Activation of For3 by the GTPase Cdc42 and Bud6 enables the nucleation of actin filaments that will be subsequently assembled into actin cables.^12^^,^^46^ Similar to the case of Mod5, the lack of Bud6 did not change the phenotype of Bgs1-depleted cells even without sorbitol (Figure S1B). When Bgs1 depletion was performed in the absence of For3, the elongated and multiseptated phenotype remained, but the cells were wider and shorter due to the additive phenotype of For3 absence (Figure S1B). Similarly, Bgs1 depletion in the absence of either the polarity marker Tea3, which facilitates Tea1 anchoring to the plasma membrane of the non-growing pole,^47^ Cdc42 function in the *cdc42-D76G* and *cdc42-L160S* hypomorphic mutants, or Cdc42 effector kinases Shk1 and Shk2,^48^ did not promote the loss of growth polarity and rounded morphology. These cells maintained the elongated and multiseptated phenotype even without sorbitol (Figure S1B; Table 1).

We also analyzed single, double, and triple deletions of *mod5*Δ, *bud6*Δ, and *for3*Δ together with deletions of *tea1*Δ and *tea4*Δ, and in no case was the formation of spherical cells observed, even under the strongest conditions with triple deletions (Figure S1C; Table S1). These results suggest that (i) Bgs1 cooperates exclusively with the Tea1-Tea4 complex, but not with the rest of polarisome, in the regulation of the growing poles, and (ii) the absence of actin cables, owing to polarisome deletions, is not involved in the loss of growth polarity observed during *bgs1*^+^ repression in the absence of Tea1 or Tea4.

### The polarized localization of Tea1, Tea4, and glucan synthases is not affected in the corresponding absence of Tea1, Tea4, or Bgs1

The previous results showed that only Bgs1 and the Tea1-Tea4 complex are involved in the control of polarized growth. Then, we examined if the absence of Bgs1 or Tea1-Tea4 alters the polarized localization of each other, and of the other glucan synthases Bgs4 and Ags1. The *tea1*Δ and *tea4*Δ deletion mutants fail to reinstate polarized growth along the long axis of the cell, activating a new incorrectly positioned growing site to form bent and T-shaped cells.^9^^,^^10^ In these mutants, Bgs1, Bgs4, and Ags1 appeared correctly polarized to the ectopic new growing tips (Figure S2A, arrow). Next, the location of Tea1, Tea4, and the other glucans synthases Ags1 and Bgs4 during *bgs1*^+^ repression, was analyzed. Cells expressing *bgs1*^+^ (0 h of repression) showed Tea1 and Tea4 localized to both growing and non-growing poles even during cytokinesis (Figure S2B, upper panels), while Bgs4 and Ags1 localized to only the growing poles during interphase and to the septum during cytokinesis (Figure S2B, lower panels). In a time course analysis of *bgs1*^+^ repression, Tea1 and Tea4 remained localized to all the poles after 24 and 36 h of repression (Figure S2B, upper panels). Similarly, Bgs4 and Ags1 remained correctly localized to the growing poles after 24 and 36 h of *bgs1*^+^ repression. However, because during *bgs1*^+^ repression most of the cells evolve to multiseptated being in the cytokinesis stage, Bgs1 and Ags1 mostly localized to the septum and some to the cytoplasm during the interphase-cytokinesis transition (Figure S2B, lower panels). Therefore, the polarized growth is not affected, and Tea1, Tea4, Bgs1, and the other glucan synthases, remain correctly localized in the corresponding absence of Tea1, Tea4, or Bgs1.

### The simultaneous absence of Bgs1 and Tea1-Tea4 complex suppresses the polarized location of the other glucan synthases Bgs4 and Ags1

**Figure 2:**
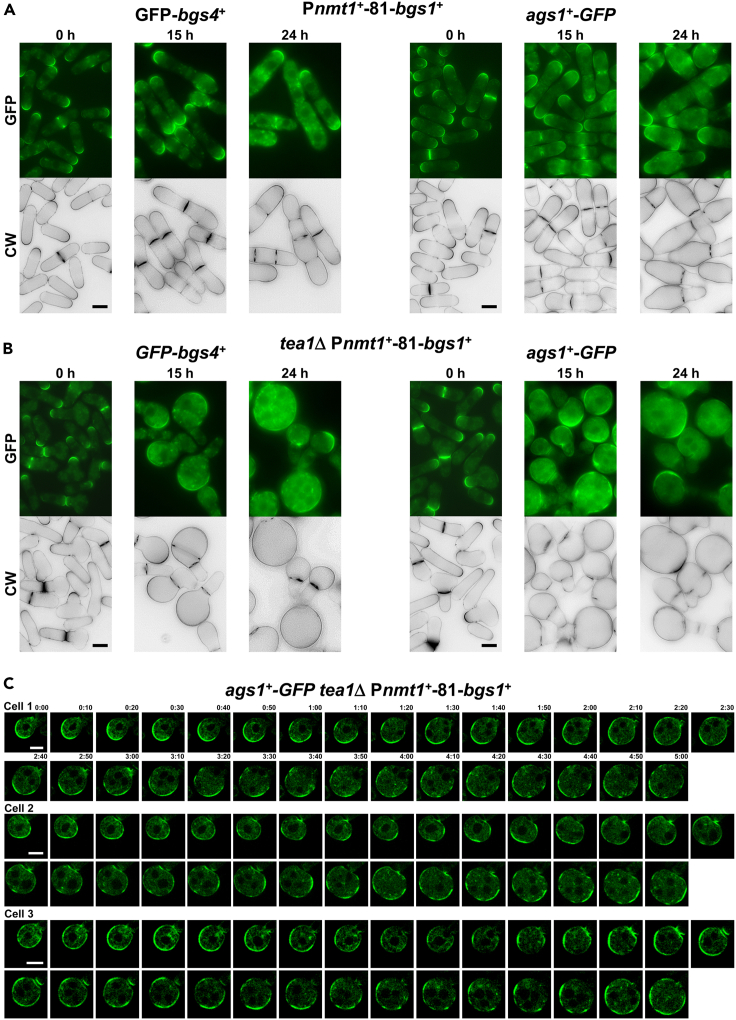
The joint absence of Bgs1 and Tea1 alters the location of the other glucan synthases Bgs4 and Ags1 (A and B) Fluorescence micrographs of CW-stained P*nmt1*^*+*^-81-*bgs1*^+^ (A) and *tea1*Δ P*nmt1*^*+*^-81-*bgs1*^+^ (B) cells carrying either *GFP-bgs4*^*+*^ (left panels) or *ags1*^*+*^*-GFP* (right panels). Cells were grown to early log-phase at 28°C in MM, transferred to MM + T, and imaged for CW and GFP fluorescences using time course microscopy at the indicated times with thiamine (0, 15, and 24 h). (C) Time-lapse sequences from the onset of formation of spherical cells and spreading of Ags1-GFP (cell 1) to create enlarged rounded cells maintaining Ags1-GFP around the cell. Early log-phase *ags1*^*+*^*-GFP tea1*Δ P*nmt1*^*+*^-81-*bgs1*^+^ cells grown at 28°C in MM were transferred to MM + T for 15 h and imaged for 5 h through GFP fluorescence time-lapse video microscopy. Elapsed time is shown in hours and minutes. Scale bars, 5 μm. See also Figures S2 and S3.

During cytokinesis, Bgs1 is required for the proper concentration of Bgs4 and Ags1 glucan synthases at the division site and collaborates with paxillin Pxl1 and calcineurin in correctly maintaining the glucan synthases in the septum membrane during cytokinesis.^31^^,^^32^^,^^33^^,^^36^ We aimed to investigate whether a similar collaboration of Bgs1 with the Tea1-Tea4 complex is necessary for maintaining the polarized localization of Bgs4 and Ags1 at the growing poles. To this end, the localization of GFP-Bgs4 or Ags1-GFP during *bgs1*^*+*^ repression, either in the presence (Figures 2A and S3A) or absence of Tea1 or Tea4 (Figures 2B and S3A), was analyzed. In cells with Bgs1 (0 h of repression), GFP-Bgs4 and Ags1-GFP localized to the growing poles and septum either in the presence or absence of Tea1 or Tea4 (Figures 2A, 2B, and S3A). After 15 and 24 h of *bgs1*^*+*^ repression in the presence of Tea1 or Tea4, GFP-Bgs4, and Ags1-GFP localization remained restricted to the cell poles and septum (Figures 2A, S2B, and S3A). The same depletion of Bgs1 in the absence of Tea1 or Tea4 led to the formation of rounded cells. In these cells, GFP-Bgs4 and Ags1-GFP lost their restricted localization, appearing in domains heterogeneously distributed throughout the plasma membrane of the spherical cells (Figures 2B and S3A). Subsequently, the localization of glucan synthases from the onset of formation of spherical cells to enlarged rounded cells was analyzed by time-lapse microscopy in *tea1*Δ P*nmt1*^*+*^-81-*bgs1*^+^ cells. The localization of Ags1-GFP was examined during the interval from 15 h (time 0:00, incipient rounded cell) to 20 h (time 5:00, enlarged rounded cell) of *bgs1*^+^ repression in the absence of Tea1 (Figures 2C and S3B). Ags1-GFP spread throughout the plasma membrane of the incipient rounded cells after 15 h, covering the entire membrane or large areas that moved over time to other sites even after 20 h of repression, thus contributing to the formation and maintenance of the spherical phenotype (Figures 2C and S3B). In cells that still had not completely lost their rod shape, Ags1-GFP extended from the pole before the start of developing rounded morphology and then, continued spreading around the membrane as the cells became rounded (Figure 2C cell 1, and S3B cells 1, 2, and 3). This suggests that the Ags1-GFP mislocalization is a consequence of the loss of growth polarity, causing glucan synthases spread and isotropic growth, and not the opposite. Therefore, the rounded morphology is not due to the loss of glucan synthases and weak cell wall formation. Overall, these results show that the isotropic growth observed upon Bgs1 depletion in cells lacking Tea1 or Tea4 is due to the altered localization all over the membrane of Bgs4 and Ags1, the enzymes that synthesize the major cell wall polymers.

### Bgs4 and Ags1 glucan synthases do not participate in the collaboration with the Tea1-Tea4 complex that regulates growth polarity

Bgs4 is the catalytic subunit of the β(1,3)glucan synthase that synthesizes branched β(1,3)glucan, the most abundant and essential polysaccharide in the cell wall,^21^^,^^25^ and Ags1/Mok1 synthesizes α(1,3)glucan, another major and essential cell wall polysaccharide.^22^^,^^23^^,^^24^ Like Bgs1, Bgs4, and Ags1 are essential and localize to the growing poles during interphase and to the division site during cytokinesis. To investigate if these enzymes also cooperate with the Tea1-Tea4 complex in the regulation of growth polarity, we analyzed the depletion of Bgs4 or Ags1 in cells lacking Tea4 at the longest repression time just during the emergence of cell lysis. To delay and partially protect the cells from the lysis phenotype caused by the absence of Bgs4 or Ags1, the cells were grown with sorbitol, delaying the appearance of cell lysis until 8–10 h of thiamine repression, as reported in previous time-course studies of *bgs4*^*+*^ and *ags1*^*+*^ repressions.^21^^,^^22^^,^^25^ Repression of *tea4*Δ P*nmt1*^*+*^-81-*bgs4*^+^ and *tea4*Δ P*nmt1*^*+*^-81-*ags1*^+^ cells for 9 h and 7 h, respectively, did not cause any additional morphological phenotype compared to the same *bgs4*^*+*^ and *ags1*^*+*^ repressions in cells with Tea4 (Figure S3C). The examination of longer repression times was not possible due to the reported physical limitation of cell lysis caused by the strong cell wall decrease.^22^^,^^25^ Therefore, the glucan synthase role cooperating with the Tea1-Tea4 complex in the control and maintenance of growth polarity is unique to the Bgs1 isoform.

Given that the localization of the other glucan synthases contributes to the isotropic growth promoted by Bgs1 depletion in the absence of the Tea1-Tea4 complex and considering that the single defect in the other glucan synthases promotes rounded morphology due to a cell wall weakness, we analyzed the possible suppression of rounded growth polarity defects by overproducing the other glucan synthases in Bgs1-depleted cells lacking either Tea1 or Tea4. In no case did the overproduction of Bgs4 or Ags1 suppress the spherical phenotype promoted by Bgs1 depletion without Tea1 or Tea4 (Figure S3D). This suggests that no other glucan synthase can compensate for the polarized growth defect resulting from the absence of Bgs1 and Tea1-Tea4 complex.

### Bgs1 loss of function, with Bgs1 mutant protein remaining correctly localized, causes the same loss of growth polarity as that of Bgs1 depletion

**Figure 3:**
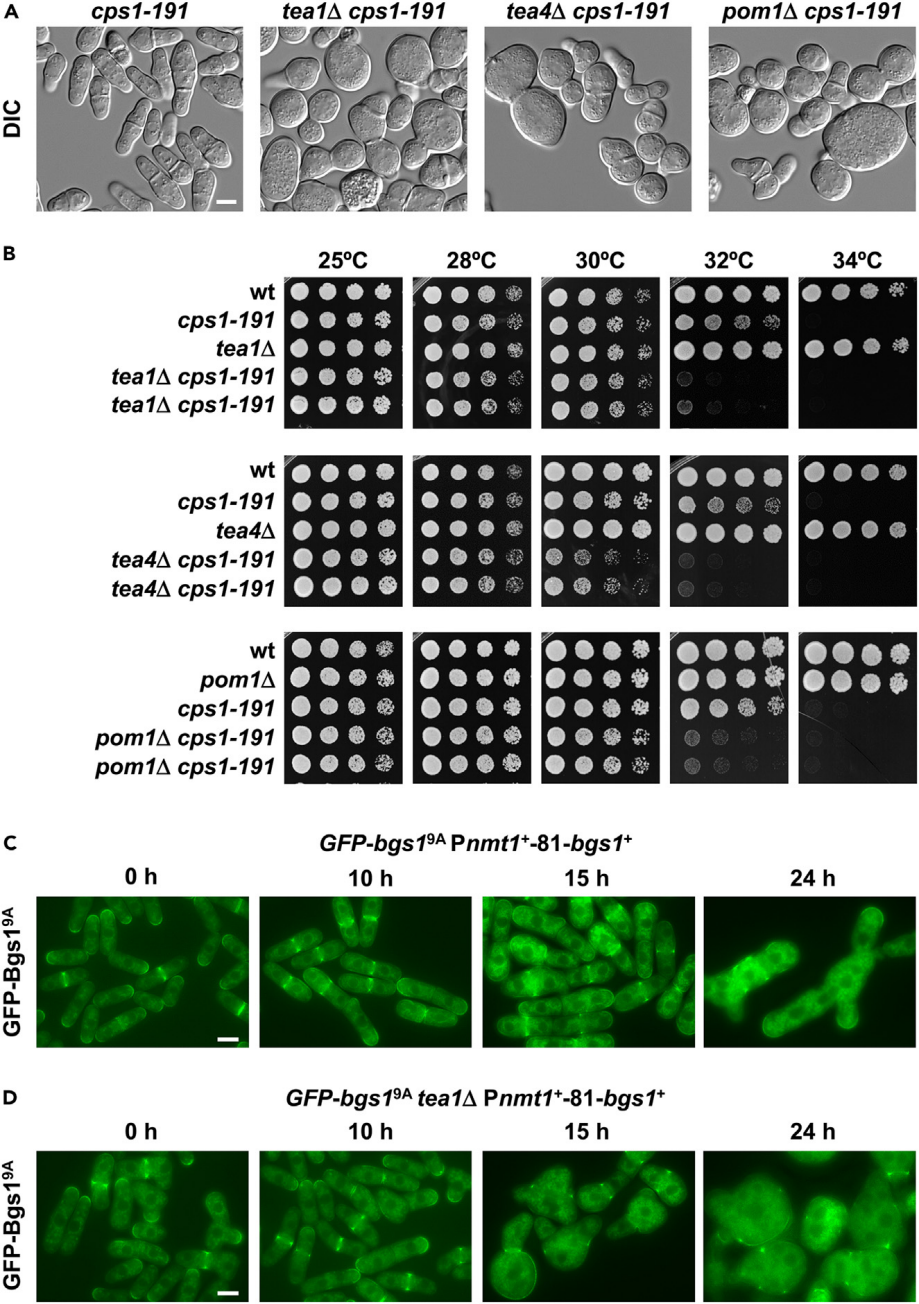
Bgs1 loss of function, with the GFP-Bgs1 mutant protein remaining correctly localized, in the absence of Tea1 causes the same loss of growth polarity phenotype as that of simultaneous absence of Bgs1 and Tea1 (A) Differential interference contrast (DIC) micrographs showing the morphology of early log-phase *cps1-191*, *tea1*Δ *cps1-191*, *tea4*Δ *cps1-191*, and *pom1*Δ *cps1-191* mutant cells grown at 25°C in YES medium, shifted to 30°C for 24 h, and imaged. *cps1-191* is a thermosensitive mutant allele of *bgs1*^*+*^. (B) Cell growth of wild-type (WT) and *cps1-191*, *tea1*Δ, and *tea1*Δ *cps1-191* mutant cells in YES solid medium at different temperatures (25, 28, 30, 32°C, and 34°C). Cells were spotted (5 μL, A_600_ of 1.0) at 1/4 dilutions on YES agar plates and grown for 3 days. (C and D) Time course fluorescence micrographs of P*nmt1*^*+*^-81-*bgs1*^+^ (C) and *tea1*Δ P*nmt1*^*+*^-81-*bgs1*^+^ (D) cells carrying the non-functional correctly localized Bgs1^9A^ mutant version tagged with GFP (*GFP-bgs1*^*9A(1136-1144)*^). Cells were grown at 28°C in MM, transferred to MM + T, and imaged for GFP fluorescence using time course microscopy at the indicated times with thiamine (0, 10, 15, and 24 h). Scale bars, 5 μm. See also Figures S3 and S4.

Bgs1 is an integral membrane protein with 14 putative transmembrane domains separated by a central cytosolic region (http://wlab.ethz.ch/protter/#up=FKS1_SCHPO&tm=auto).^49^ This cytosolic region exhibits homology with other glycosyltransferases and contains residues potentially involved in binding to the substrate UDP-glucose and consequently, in catalytic activity.^37^^,^^50^^,^^51^^,^^52^ To discern whether the presence of Bgs1 itself or its function is responsible for regulating the growth polarity in collaboration with the Tea1-Tea4 complex, we analyzed the *cps1-191* mutant strain alone, which carries a thermosensitive allele of *bgs1*^*+*^,^53^ and with the *tea1*Δ, *tea4*Δ, or *pom1*Δ deletions. The original *cps1-191* mutant strain carries an additional S338N mutation in the Mto2 sequence.^54^ We confirmed that all the examined single and double *cps1-191* mutant strains contain the WT *mto2*^*+*^ sequence (see STAR Methods). The *cps1-191* mutant presents after long times at high restrictive temperature (8 h at 36°C) different morphological defects, mainly oval, elongated, and a few rounded cells, but never a general phenotype of spherical cells.^53^ However, at the permissive temperature of 30°C the single *cps1-191* mutant strain displayed almost normal morphology, whereas all double mutant strains presented misshapen and rounded morphology (Figure 3A). Moreover, these double mutants exhibited poor growth at temperatures above 30°C, while the parental mutant strains were able to grow at least up to 32°C (Figure 3B). As observed above with Bgs1 depletion (Figure S3D), overproduction of Bgs4 or Ags1 in the *cps1-191* strains was unable to suppress the spherical phenotype promoted by Bgs1 loss of function in the absence of Tea1 or Tea4 (Figure S3E). These results suggest the involvement of Bgs1 function, and therefore its linear β(1,3)glucan, in the control of growth polarity in collaboration with the Tea1-Tea4 complex.

To confirm the specific involvement of Bgs1 function and its linear β(1,3)glucan in the regulation of growth polarity in collaboration with the Tea1-Tea4 complex, we analyzed a special non-functional correctly localized mutant version of GFP-Bgs1 named GFP-Bgs1^9A(1136-1144)^, where the 9-amino acid residues 1136 to 1144 of the cytosolic region containing the putative catalytic site of Bgs1 were replaced by 9 alanines (Figure S4). Other GFP-Bgs1^9A^ mutants displayed the typical patterns, either functional correctly localized like GFP-Bgs1^9A(10-18)^ or non-functional non-localized like GFP-Bgs1^9A(334-342)^ (Figure S4). These *GFP-bgs1*^*9A*^ sequences were inserted into the P*nmt1*^*+*^-81-*bgs1*^+^ strain (see STAR Methods). The resulting strains contained two copies of *bgs1*, a *GFP-bgs1*^*9A*^ expressed under its own promoter, and a WT *bgs1*^+^ expressed under the control of the *nmt1*^*+*^-81 promoter (Figure S4). Typical 9-alanine constructs with correctly localized GFP-Bgs1^9A^ to poles and septum when additional *bgs1*^*+*^ was induced, like GFP-Bgs1^9A(10-18)^, proved to be functional because they maintained WT cell phenotype and correct GFP-Bgs1^9A^ localization after repression of additional *bgs1*^*+*^. This pattern was similar to the localization and WT cell phenotype of positive control strain with GFP-Bgs1 during both induction and repression of additional *bgs1*^*+*^, and opposite to the elongated and multiseptated phenotype displayed after repression of a negative control strain with only P*nmt1*^*+*^-81-*bgs1*^+^ without additional GFP-Bgs1 copy (Figures S4A, S4B, and S4C). Other typical 9-alanine constructs that proved to be non-functional because displayed elongated and multiseptated phenotype after additional *bgs1*^*+*^ repression, like GFP-Bgs1^9A(334-342)^, exhibited non-localized GFP-Bgs1 distribution under both *bgs1*^*+*^-induced and *bgs1*^*+*^-repressed conditions (Figure S4D). P*nmt1*^*+*^-81-*bgs1*^+^ cells containing GFP-Bgs1^9A(1136-1144)^ showed the same elongated and multiseptated *bgs1*^+^-repression phenotype (Figures 3C and S4E) as that of control P*nmt1*^*+*^-81-*bgs1*^+^ cells without additional GFP-Bgs1 copy (Figure S4A), indicating that this GFP-Bgs1^9A(1136-1144)^ version is non-functional. However, this non-functional GFP-Bgs1^9A(1136-1144)^ version proved to be unique because it remained localized to the growing sites and septum in both additional *bgs1*^*+*^-induced and *bgs1*^*+*^-repressed cells. During the generation of the elongated and multiseptated repression phenotype, the GFP-Bgs1^9A(1136-1144)^ fluorescence level decreased slightly at the plasma membrane and increased inside the cell (Figures 3C and S4E), similar to that of GFP-Bgs4 and Ags1-GFP in multiseptated *bgs1*^*+*^-repressed cells (Figures 2A and S3A).^32^^,^^33^ Thus, this GFP-Bgs1^9A(1136-1144)^ construct is non-functional but able to stay correctly localized to the growing poles and septum even in the absence of a functional Bgs1 copy (Figures 3C and S4E). However, the cells lacking Tea1 became rounded after 15 h of depletion of additional Bgs1 copy (100% rounded cells), and the non-functional correctly localized GFP-Bgs1^9A(1136-1144)^ spread throughout the plasma membrane and also increased inside the cells like in cells with Tea1 (Figures 3C and 3D). Therefore, the correct localization of non-functional Bgs1^9A(1136-1144)^ do not prevent the loss of growth polarity in cells lacking Tea1, revealing an essential role of Bgs1-dependent linear β(1,3)glucan for the control and maintenance of growth polarity, as it has been shown for cytokinesis.^28^

### The absence of Bgs1 function, with inactive Bgs1 protein remaining localized, results in diffusion of sterols and delocalization of actin patches all over the plasma membrane

**Figure 4:**
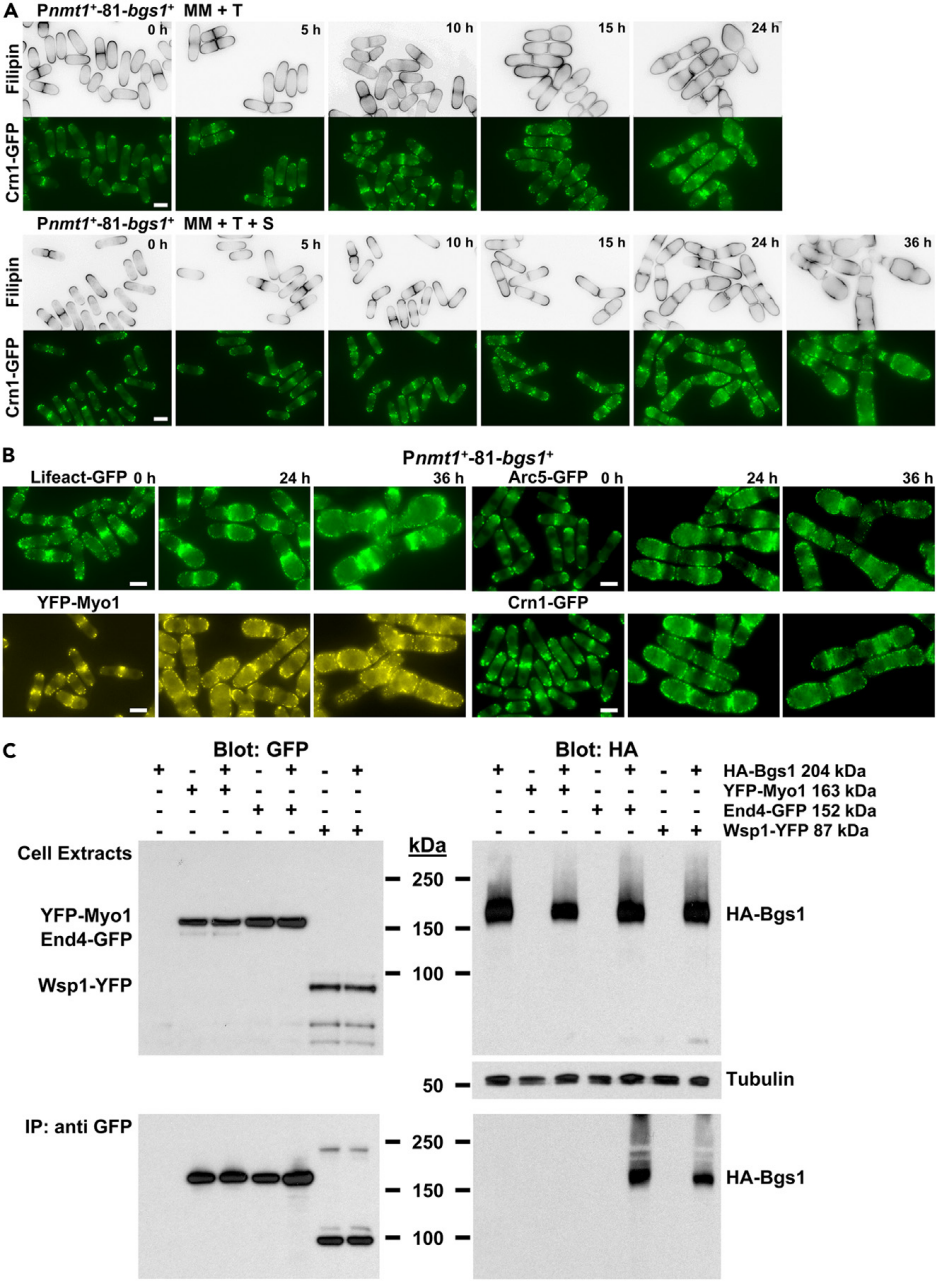
Bgs1 controls the localization of sterol-rich membrane (SRM) domains and actin patches, and physically interacts with the actin patch components Wsp1 and End4, but not with Myo1 (A) Time course fluorescence micrographs of filipin-stained P*nmt1*^*+*^-81-*bgs1*^+^ cells carrying *crn1*^*+*^*-GFP*. Early log-phase cells grown at 28°C in MM (upper panels) or MM + S (lower panels) either in the absence or presence of thiamine, were stained with filipin to observe sterol-rich membrane (SRM) domains and imaged for filipin and coronin Crn1-GFP fluorescences using time course microscopy at the indicated times with thiamine (0, 5, 10, 15, and 24 h for MM + T, and the same times plus 36 h for MM + T + S). (B) Fluorescence micrographs of P*nmt1*^*+*^-81-*bgs1*^+^ cells carrying either *Lifeact-GFP*, *arc5*^*+*^*-GFP*, *YFP-myo1*^*+*^, or *crn1*^*+*^*-GFP* to visualize actin patches. Cells were grown to early log-phase at 28°C in MM + S, transferred to MM + T + S, and imaged for GFP or YFP fluorescence using time course microscopy at the indicated times with thiamine (0, 24, and 36 h). Scale bars, 5 μm. (C) Extracts of cells expressing HA-Bgs1 and either YFP-Myo1, End4-GFP, or Wsp1-YFP were immunoprecipitated with polyclonal anti-GFP antibodies, and the precipitates were probed with monoclonal anti-HA or anti-GFP antibodies. Expression of YFP-Myo1, End4-GFP, Wsp1-YFP and HA-Bgs1 in cell extracts was analyzed by western blot with monoclonal anti-GFP or anti-HA antibodies. The extracts were also probed with monoclonal anti-α-tubulin antibody as the loading control. See also Figures S5 and S6.

SRM domains define prospective growth sites in *S. pombe*, whereas the Tea1-Tea4 complex and the F-actin cytoskeleton, in particular actin patches, provide different mechanisms that collaborate in the restriction of SRM domains to the growth areas.^7^ In agreement, *myo1*Δ *tea1*Δ cells, with double deletion of the actin patch component myosin-I Myo1 and the polarity marker Tea1, present enlarged and mispositioned growth sites relative to the long cell axis, and SRM domains localize throughout the plasma membrane but enriched at the growth sites, suggesting the existence of Myo1 and Tea1 redundant mechanisms for confining growth, and a Myo1 and Tea1-independent growth and SRM domains confining system.^7^ As we described here, Bgs1 cooperates specifically with Tea1-Tea4, but not with other polarisome proteins, in the actin cables-independent control and maintenance of growth polarity. Therefore, we considered that Bgs1 function could influence this independent SRM domains organization for confining growth. To investigate this hypothesis, SRM domains were analyzed in P*nmt1*^*+*^-81-*bgs1*^+^ cells during *bgs1*^+^ repression either in the absence or presence of sorbitol. The cells were stained with filipin, a dye that stains sterols in the fungal plasma membrane, and examined through time course microscopy. In the presence of Bgs1, SRM domains appeared polarized in both poles and septum area (Figure 4A, 0 h). In contrast, depletion of Bgs1 promoted, after 10 h without and after 15 h with sorbitol, the spread and uniform staining of sterols all over the plasma membrane, long before the appearance of the morphological *bgs1*^+^-repression phenotype (Figure 4A, 15 h and 24 h, respectively), and although polarized growth was maintained during all the time course (Figure 4A, 24 h and 36 h, respectively). To corroborate this sterol dependence on Bgs1, P*nmt1*^*+*^-81-*bgs1*^+^ cells expressing the sterol biosensor D4H fused to Cherry-RFP,^55^ were examined during *bgs1*^+^ repression through time course microscopy (Figure S5A). Control cells with induced Bgs1 presented a heterogeneous D4H localization to the plasma membrane; different from that of filipin as reported.^55^ Like filipin, after 10 h of *bgs1*^+^ repression D4H started to spread along the plasma membrane, long before the appearance of morphological changes, and after 15 h and 24 h, it localized all over the plasma membrane (Figure S5A). This indicates that Bgs1 is required for the correct localization and maintenance of SRM domains in the growth sites, and that Bgs1 requirement is more critical for the maintenance of SRM domains than of WT morphology.

Because sterols extend throughout the membrane when actin is depolymerized or actin patches are defective,^7^^,^^56^ and because sterols also spread when Bgs1 is absent, we analyzed the actin patches cytoskeleton of cells depleted of Bgs1. Repression of *bgs1*^+^ analyzed through time course in a strain carrying the actin patch-associated coronin Crn1,^57^ caused actin patches depolarization, but not their depolymerization, after 10 h of repression (or 15 h with sorbitol), coincident with the start of sterols spread and earlier than the onset of morphological defects after 15 h of repression (or 24 h with sorbitol) (Figures 4A and S5A). At longer times, the actin patches appeared like sterols, distributed throughout the whole-cell periphery independently of the growth sites (Figures 4A and S5A). This was corroborated by analyzing the localization of the actin patch components actin-probe Lifeact, Arp2/3 subunit Arc5, myosin-I Myo1, coronin Crn1, plasma membrane clathrin adaptor End4/Sla2, and Wiskott-Aldrich syndrome protein Wsp1^57^^,^^58^^,^^59^^,^^60^^,^^61^ after different times of *bgs1*^+^ repression (Figures 4B and S5B). The same result was also obtained in both single sections and maximal intensity projections of P*nmt1*^*+*^-81-*bgs1*^+^ cells stained with Alexa Fluor 448-phalloidin to detect actin patches after different times of *bgs1*^+^ repression (0, 24, and 36 h) (Figure S5C). Globally, these results indicate that Bgs1 regulates the polarization of SRM domains and actin patches. Consequently, Bgs1 depletion promotes both the spread of sterols and the depolarization of actin patches all over the plasma membrane independently of the growth sites, indicating that SRM domains and actin patches depend on Bgs1 to define the prospective growth sites.

The dispersion of actin patches caused by depletion of Bgs1 prompted us to analyze if there is a physical interaction between Bgs1 and actin patches by co-immunoprecipitation experiments using a strain containing HA-Bgs1 and either End4-GFP, Wsp1-YFP, or YFP-Myo1.^59^^,^^60^^,^^61^ The experiments showed a clear specific interaction between Bgs1 and some actin patch components, End4 and Wsp1, but not between Bgs1 and Myo1 (Figure 4C). The fact that Bgs1 is a large integral plasma membrane protein suggests that the Bgs1 control on actin patches to keep them polarized in the growth sites occurs at the plasma membrane by interacting with specific actin patch components.^62^^,^^63^^,^^64^

To examine the possible involvement of Bgs1 function in the polarization of sterols and actin patches, we analyzed the distribution of SRM domains and actin patches in the thermosensitive *cps1-191* strain grown at 25°C and 34°C for 4 h, at the onset of thermosensitive phenotype (Figure S5D). Filipin staining showed polarized SRM domains in WT and *cps1-191* cells grown at 25°C, but starting to spread throughout the plasma membrane of *cps1-191* cells after 4 h at 34°C. Similarly, visualization of Lifeact-GFP showed polarized actin patches in WT and *cps1-191* cells grown at 25°C, and the start of actin patches depolarization in *cps1-191* cells after 4 h at 34°C (Figure S5D). These results suggest that Bgs1 function is involved in the polarized localization of both SRM domains and actin patches.

To confirm the essential role of Bgs1 function in polarization of sterols and actin patches, we also analyzed through time course the localization of both SRM domains and actin patches in the presence of the non-functional correctly localized Bgs1^9A(1136-1144)^ mutant (Figure S5E). Repression of *bgs1*^+^ in a strain carrying simultaneously the non-functional Bgs1^9A(1136-1144)^, induced both the spread of sterols and depolarization of actin patches throughout the membrane after 10 h of Bgs1 depletion (Figure S5E). These results show that the localization of Bgs1 to the plasma membrane is necessary but not sufficient, and that the linear β(1,3)glucan it produces is essential to maintain the polarized localization of both SRM domains and actin patches in fission yeast.

### Bgs1 localization is not dependent on either actin polymerization or SRM domains polarization

Subsequently, we investigated whether actin patches and SRM domains regulate in turn Bgs1 localization. Cells carrying *RFP-bgs1*^*+*^ were treated for 3 h with high concentrations of latrunculin A (LatA, 100 μM), capable of causing actin patches depolymerization in less than 5 min,^33^ stained with filipin, and observed using time course microscopy. F-actin depolymerization with LatA led after 1 h to the dispersion of sterols all over the membrane (Figure S6A), whereas Bgs1 remained localized to the growing poles for at least 3 h (Figure S6A), as reported for Bgs4.^21^ Therefore, Bgs1 localization is not dependent on either actin polymerization or SRM domains polarization. However, SRM domains polarization is dependent on actin polymerization.

It has been reported that prolonged treatment with filipin causes alterations in SRM domains.^6^^,^^65^ To corroborate this independent localization of Bgs1 on SRM domains, *RFP-bgs1*^*+*^ *crn1*^*+*^*-GFP* cells were treated for 1 h with filipin (5 μg/mL) to alter the sterols organization, and observed through time course microscopy (Figure S6B, upper panels). After 30 min with filipin, sterols spread along the plasma membrane (Figure S6B, upper panels). However, Bgs1 glucan synthase and actin patches remained localized to poles and septum throughout the filipin treatment (Figure S6B, upper panels). Similar independent localization of Bgs4 on SRM domains was confirmed in *GFP-bgs4*^*+*^ cells treated for 1 h with filipin (Figure S6B, lower panels). This indicates that glucan synthases and actin patches localizations are not dependent on SRM domains polarization.

### Actin patches polarization is responsible for the control of growth polarity with the Tea1-Tea4 complex

**Figure 5:**
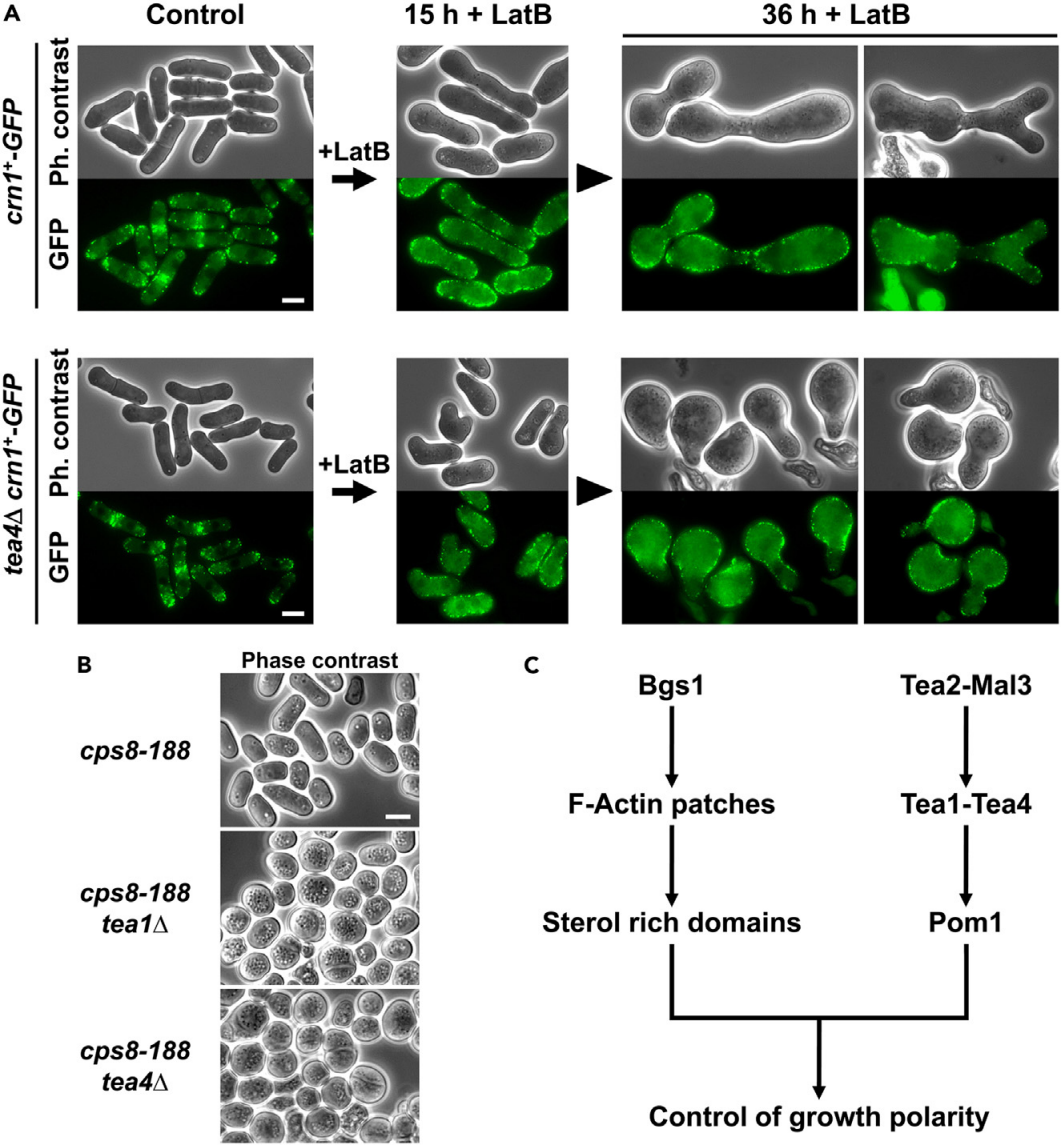
Actin patches polarization is responsible for the control of growth polarity together with the Tea1-Tea4 complex (A) Phase contrast and GFP fluorescence micrographs of WT and *tea4*Δ cells carrying *crn1*^*+*^*-GFP*. Cells were grown at 28°C in YES medium (control), then a low concentration of Latrunculin B (LatB, 20 μM) to cause the delocalization but not depolymerization of actin patches was added, and the cells were imaged for phase contrast and GFP fluorescence using time-course microscopy at the indicated times with LatB (0, 15, and 36 h). (B) Phase contrast micrographs showing the rod shape or rounded morphology of *cps8-188*, *tea1*Δ *cps8-188*, and *tea4*Δ *cps8-188* mutant cells. Cells were grown to stationary phase at 28°C on MM plates for 8 days and imaged. *cps8-188* is a thermosensitive mutant allele of the actin gene *act1*^*+*^. Scale bars, 5 μm. (C) Scheme of the essential cooperation between Bgs1 and the specific Tea1-Tea4 complex in the control and maintenance of growth polarity. See also Figures S5–S7.

It has been proposed that the absence of Myo1 alters the polarized growth in cells lacking Tea1 due to the requirement of functional actin patches for the endocytosis of sterols in non-growing areas.^7^ In this sense, our observations revealed that the absence or loss of function of different actin patch components including Myo1, led to the loss of growth polarity in Tea1-deleted cells (Figure S7A). The absence of Myo1, or Wsp1, an activator of the actin nucleator complex Arp2/3 ^61^, as well as the loss of function of Arp3 (thermo- and cold-sensitive mutant *arp3-C1*), the actin-like subunit of this complex,^66^ induced a rounded morphology in cells lacking Tea1 (Figure S7A). In contrast, under the same growth conditions, control *myo1*Δ, *wsp1*Δ, and *arp3C-1* mutant cells containing Tea1, maintained rod-shape morphology (Figure S7A). Intriguingly, repression of *bgs1*^+^ in *wsp1*Δ or *arp3-C1* cells did not cause loss of growth polarity (Figure S7B). A similar dual pattern was observed when WT or *tea4*Δ cells were grown with low concentrations of latrunculin B (LatB, 20 μM). This specific low concentration of LatB caused the actin patches delocalization around the cell periphery, but not their depolymerization. Under these conditions, in which actin patches were still present but depolarized, *tea4*Δ, but not WT cells, exhibited a complete loss of growth polarity and rounded morphology (Figure 5A). Similar to LatA, a higher dosage of LatB caused total actin patch depolymerization,^33^ while a lower dosage of LatB resulted in poor or no delocalization of actin patches at all. In both cases, no effect on the loss of growth polarity in the absence of Tea4 was observed (data not depicted). The actin mutant *cps8-188* exhibits cells in which actin remains polymerized but randomly distributed, and the contractile actomyosin ring is absent.^67^ Correspondingly, cells of the *cps8-188* mutant showed a complete loss of growth polarity and rounded morphology in the absence of Tea1 or Tea4, but displayed normal growth polarity and elongated morphology in their presence (Figure 5B). Therefore, both the absence of Bgs1 function and the defective actin patches localization caused a similar total loss of growth polarity in the absence of the Tea1-Tea4 complex. Altogether, these observations suggest that Bgs1 is necessary for the correct localization of actin patches and SRM domains, which, in turn, are ultimately responsible for the control of growth polarity along with the Tea1-Tea4 complex (Figure 5C).

### Bgs1 is required for endocytosis and membrane traffic

**Figure 6:**
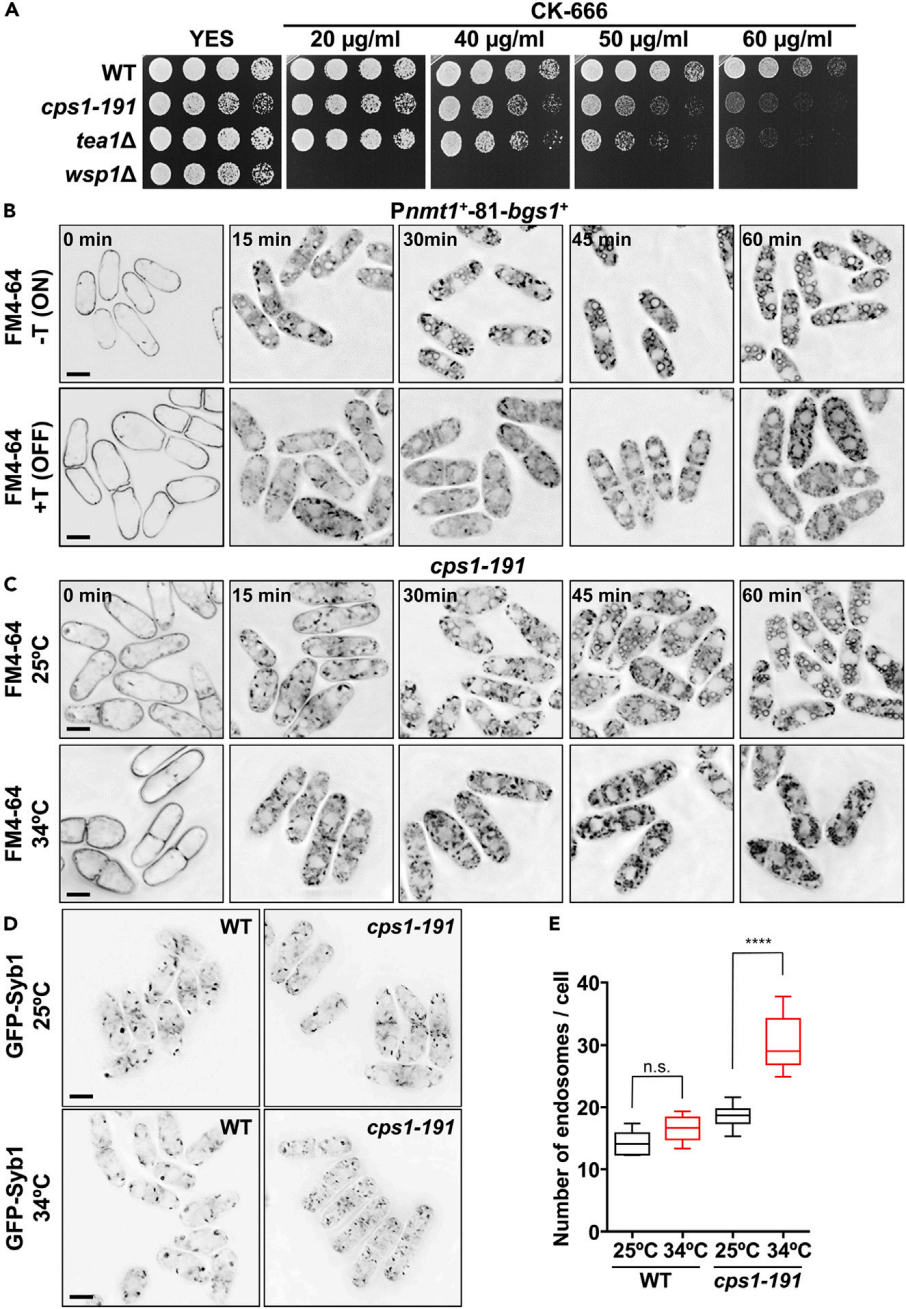
Bgs1 is required for endocytosis and the correct formation of endosomes and vacuoles (A) Cell growth of WT, *cps1-191*, *tea1***Δ,** and *wsp1*Δ cells in YES solid medium with different concentrations of the Arp2/3 complex inhibitor CK-666. Cells were spotted (5 μL, A_600_ of 1.0) at 1/4 dilutions on YES agar plates and grown for 3 days at 30°C. (B) Kinetics of FM4-64 internalization in P*nmt1*^*+*^-81-*bgs1*^+^ cells grown at 28°C in MM (*bgs1*^+^ ON, -T, upper panels) and transferred to MM + T for 15 h (*bgs1*^+^ OFF, +T, lower panels). The cells were imaged for FM4-64 fluorescence using time-course microscopy at the indicated times with FM4-64 (0, 15, 30, 45, and 60 min). (C) Kinetics of FM4-64 internalization in *cps1-191* mutant cells grown in MM at permissive 25°C (upper panels) and restrictive 34°C for 4 h (lower panels). The cells were imaged as in B. (D) Fluorescence micrographs of WT and *cps1-191* mutant cells carrying the endosome marker GFP-Syb1. Cells were grown in MM to early log-phase at permissive 25°C (upper panels) and restrictive 34°C for 4h (lower panels), and imaged for GFP fluorescence microscopy. Scale bars, 5 μm. (E) Boxplot showing the quantification of the number of GFP-Syb1 labeled endosomes per cell in images as in D (*n* = 100 cells). The asterisks indicate the significant statistical difference between paired strains analyzed by the Student’s test: ∗*p* < 0.05; ∗∗*p* < 0.01; ∗∗∗*p* < 0.001; ∗∗∗∗*p* < 0.0001; n. s.: not significant (*p* > 0.05). Error bars indicate standard deviation (SD). See also Figure S8.

The requirements of Bgs1 for actin patches and SRM domains polarization suggest that Bgs1 could also be necessary for actin patch-dependent endocytosis. To explore this possibility, we first analyzed the sensitivity of *cps1-191* and *tea1*Δ mutants to the Arp2/3 complex inhibitor CK-666.^68^ The mutant strain *wsp1*Δ, which is defective in endocytosis and highly hypersensitive to CK-666, was used as a control (Figure 6A). We observed that *tea1*Δ cells are more sensitive to CK-666 than WT cells. Similarly, *cps1-191* cells grown at the higher permissive temperature of 30°C were also more sensitive to the drug (Figure 6A). Altogether, the requirement of Bgs1 for the polarized localization of both actin patches and SRM domains, its physical interaction with the actin patch components End4 and Wsp1, and the hypersensitivity of the Bgs1-defective *cps1-191* mutant to CK-666, strongly suggest that this glucan synthase might play a crucial role in regulating the endocytic process.

To further explore the potential role of Bgs1 in endocytosis, we examined if the cells depleted of Bgs1 exhibit a defective uptake of FM4-64, a fluorescent styryl dye used as a marker for the endocytic pathway from the plasma membrane to vacuoles.^48^^,^^69^ Initially, we examined the kinetics of FM4-64 internalization in WT and mutant cells after 24 h of *bgs1*^*+*^ repression, (Figure S8A). WT and P*nmt1*^*+*^-81-*bgs1*^+^ cells grown in the presence of thiamine for 24 h were transferred to medium with FM4-64 for 30 min on ice, washed, and the kinetics of FM4-64 internalization were examined using time course microscopy. At 0 min, FM4-64 was present in the plasma membrane of both WT and Bgs1-depleted cells. After 3 min, FM4-64 was already endocyted in WT cells, while in the absence of Bgs1 it remained in the plasma membrane for up to 9 min, starting some endocytosis after 12 min (Figure S8A, arrow). Subsequently, P*nmt1*^*+*^-81-*bgs1*^+^ cells grown either in the absence (-T, ON) or presence of thiamine for 15 h (+T, OFF) were transferred to medium with FM4-64, processed as described previously, and the kinetics of FM4-64 transport to vacuoles were examined through time course microscopy (Figure 6B). After 15 min, FM4-64 was observed in intermediate compartments, likely endosomes, in both strains. After 30 min, vacuoles were labeled in cells containing Bgs1 (Figure 6B, upper panels), whereas those depleted of Bgs1 exhibited hazy staining of cytoplasm and small fragmented vacuolar bodies even after 60 min of FM4-64 labeling (Figure 6B, lower panels). This kinetic analysis suggests that Bgs1 levels control membrane traffic from the plasma membrane to vacuoles. Similar results were obtained with the kinetics of FM4-64 internalization in thermosensitive *cps1-191* mutant cells grown at permissive (25°C) and non-permissive (34°C) temperatures for 4 h (Figure 6C). *cps1-191* cells grown at both temperatures incorporated FM4-64 into endosomal compartments. However, at 34°C, no vacuoles were stained even after 60 min of chase, whereas at 25°C, vacuoles were labeled after 30 min (Figure 6C). Finally, the onset of this defect in FM4-64 transport to vacuoles was analyzed through time course after 0, 5, 10, 15, and 24 h of *bgs1*^*+*^ repression (Figure S8B). Control (0 h of repression) and cells after 5 h of repression displayed vacuoles labeled with FM4-64 after 60 min of chase. However, after 10 h of repression and 60 min of chase, only small fragmented bodies were stained (Figure S8B), which is coincident with the previously described onset of sterols spread and actin patches delocalization. The coincidence in the appearance of this endocytosis defect with the start of sterols and actin patches dispersion (10 h of Bgs1 depletion) suggests that these processes are interconnected and share similar Bgs1 requirements.

### Bgs1 is required for the correct formation of endosomes and vacuoles

To delve deeper into the role of Bgs1 in membrane traffic and endocytosis, we analyzed the distribution of the endosome marker synaptobrevin, Syb1, in WT and *cps1-191* mutant cells grown at 25°C and 34°C. After 4 h at 34°C, *cps1-191* cells exhibited considerably smaller and more numerous endosomes compared to those of cells grown at 25°C or WT cells grown at either 25°C or 34°C (Figures 6D and 6E).

**Figure 7:**
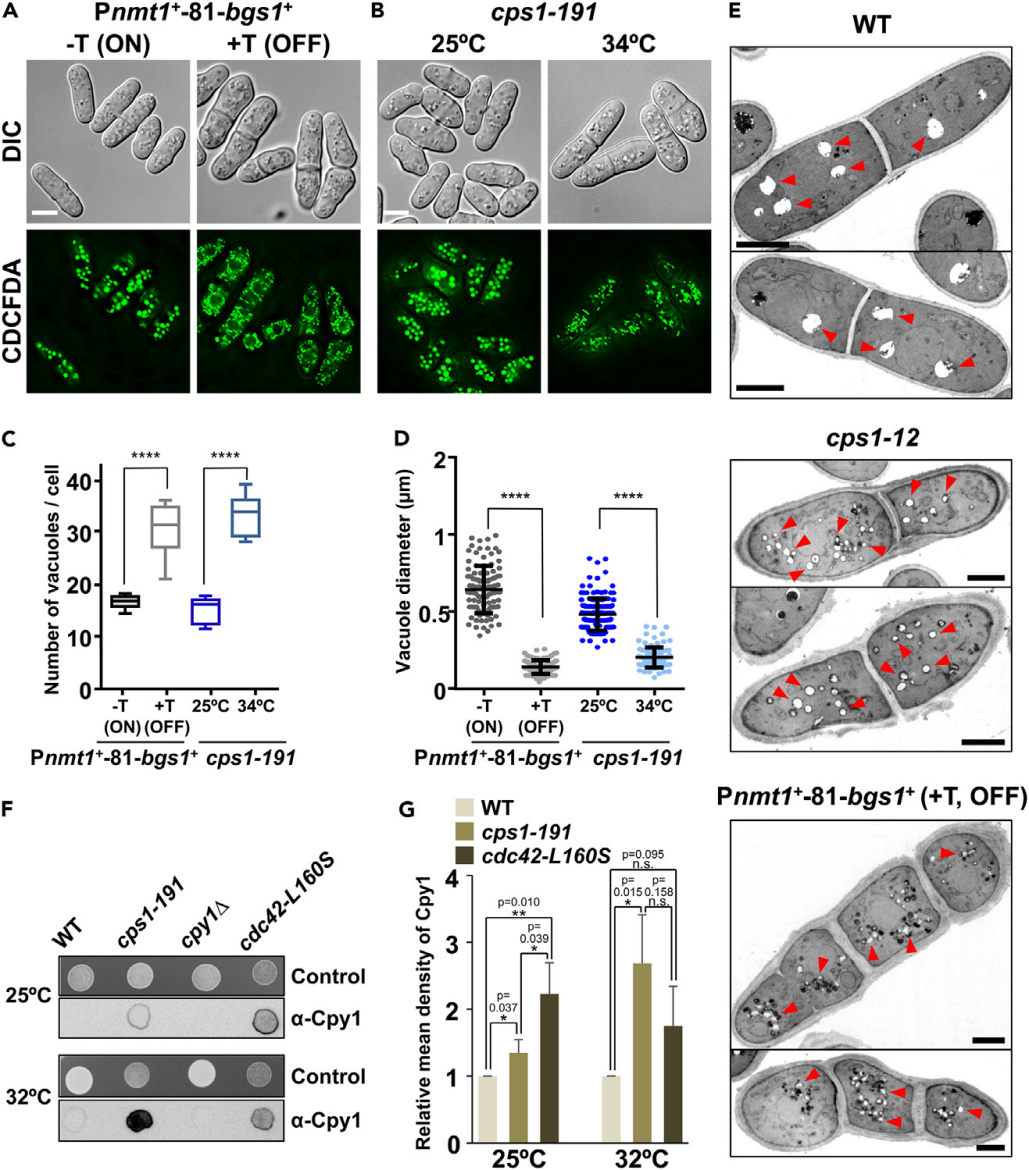
Bgs1 depletion or loss of function causes fragmented vacuoles and miss-sorting of the carboxypeptidase Y Cpy1 to the vacuole (A and B) DIC and fluorescence micrographs of CDCFDA-stained vacuoles of (A) P*nmt1*^*+*^-81-*bgs1*^+^ cells grown at 28°C in MM (*bgs1*^+^ ON, -T) and transferred to MM + T for 15 h (*bgs1*^+^ OFF, +T), and (B) *cps1-191* mutant cells grown in MM at permissive 25°C and restrictive 34°C for 4 h. Scale bars, 5 μm. (C) Boxplot showing the quantification of the number of vacuoles per cell in images as in A and B (*n* = 100 cells). (D) Quantification of the diameter of the vacuoles in images as in A and B (*n* = 100 vacuoles). The asterisks indicate the significant statistical difference between paired strains analyzed by the Student’s test: ∗*p* < 0.05; ∗∗*p* < 0.01; ∗∗∗*p* < 0.001; ∗∗∗∗*p* < 0.0001; n. s.: not significant (*p* > 0.05). (E) Transmission electron micrographs of WT (upper panels) and *cps1-12* mutant (middle panels) cells grown at 28°C in MM, and of P*nmt1*^*+*^-81-*bgs1*^+^ cells grown at 28°C in MM + T + S for 24 h (*bgs1*^+^ OFF, +T, lower panels). *cps1-12* is a thermosensitive mutant allele of *bgs1*^*+*^. Red arrowhead, vacuoles. Scale bars, 2 μm. (F) Colony immunoblot assay with anti-carboxypeptidase Y monoclonal antibody (lower panels) of WT and *cps1-191* mutant cells spotted onto nitrocellulose membranes (5 μL, A_600_ of 2.0) laid on YES agar plates, and grown at permissive 25°C and restrictive 32°C for 24 h. The *cpy1*Δ strain was included as the negative control, and the *cdc42L160S* mutant strain as the positive control. As a control for the assayed colonies, the same cells were spotted on YES agar and grown at 25°C and 32°C for 24 h (upper panels). (G) Bar chart depicting mean density values relative to WT levels of carboxypeptidase Cpy1 secretion in the colony immunoblot assays of F (mean of three independent experiments). Error bars indicate standard deviation (SD). The asterisks indicate the significant statistical difference between paired strains analyzed by the Student’s test with three (*n* = 3) independent dot blot experiments: ∗*p* < 0.05; ∗∗*p* < 0.01; n. s.: not significant (*p* > 0.05). Error bars indicate standard deviation (SD). See also Figure S8.

Additionally, we conducted CDCFDA staining to visualize the vacuoles of P*nmt1*^*+*^-81-*bgs1*^+^ cells grown either in the absence (−T, ON) or presence of thiamine for 15 h (+T, OFF) (Figure 7A). Bgs1-depleted cells displayed an increased number of small irregular vacuoles (Figures 7A, 7C, and 7D). The same vacuolar defect was observed in *cps1-191* mutant cells grown at 34°C for 4 h, whereas at 25°C presented vacuoles similar to those of WT cells (Figures 7B, 7C, and 7D). To corroborate this, the vacuolar cell lumen stained with Blue CMAC^70^ was analyzed in P*nmt1*^*+*^-81-*bgs1*^+^ cells through time course at different times of *bgs1*^*+*^ repression (Figure S8C). Control (0 h of repression) and cells after 5 h of repression exhibited similar large vacuoles. However, after 10 h of repression, the cells presented a higher number of small vacuoles (Figure S8C), coinciding in time with defects in FM4-64 transport to vacuoles and with the spread of sterols and actin patches, and long before the loss of growth polarity and onset of morphological defects (Figure S8D). Transmission electron microscopy observation of the vacuoles corroborated the observations with CDCFDA and Blue CMAC. Cells of both thermosensitive *cps1-12* grown at high permissive temperature of 28°C^50^ and P*nmt1*^*+*^-81-*bgs1*^+^ after 36 h of *bgs1*^*+*^ repression, showed smaller and more numerous vacuoles than those of WT cells (Figure 7E, red arrowhead). Altogether, these results indicate that Bgs1 function is required for the correct fusion of endosomes and vacuoles.

To further analyze the role of Bgs1 in membrane traffic, the transport of carboxypeptidase Y, Cpy1, to the vacuole, was examined. Defects in the traffic between endosomes and vacuoles can block Cpy1 sorting to the vacuole, leading to the secretion of this enzyme.^71^ We used *Saccharomyces cerevisiae* anti-carboxypeptidase monoclonal antibody to detect secreted Cpy1 in a colony immunoblot technique. The *cpy1*Δ strain was used as the negative control, and the *cdc42L-160S* mutant strain, which presents membrane traffic defects,^48^ as the positive control. We found that *cps1-191* cells grown at permissive 25°C did not exhibit abnormal secretion of Cpy1 compared to that of WT cells, whereas at restrictive 32°C, there was a strong extracellular signal (Figures 7F and 7G). Thus, this result shows that Bgs1 function is necessary for the sorting of Cpy1 to the vacuole.

## Discussion

The cells of *S. pombe* are surrounded by a polysaccharidic cell wall that provides them a very regular rod shape. Cellular growth occurs at the poles, which are determined by the microtubules-transported polarity proteins Tea1 and Tea4.^72^^,^^73^ These polarity markers contribute to the formation of SRM domains, where components required for polarized growth accumulate. It has been proposed that signaling through the Tea1-Tea4 complex stabilizes these SRM domains.^7^ On the other hand, plasma membrane endocytosis, which involves functional actin patches, is required to remove sterols from the plasma membrane and restrict the SRM domains to the growth areas. Accordingly, mutants affecting the nucleation of F-actin to form actin patches, such as *myo1*Δ, *wsp1*Δ, *arp3-C1*, or *pal1*Δ, exhibit altered polarity in the absence of the Tea1-Tea4 complex.^7^^,^^74^ Here, we show that this altered polarity eventually progresses to complete loss of growth polarity, resulting in cells totally rounded (Figure S7A).

The essential role of Bgs1 in cytokinesis has been widely studied, however, nothing is known about its possible role in growth polarity. During cytokinesis, Bgs1 is essential for polarized septum growth and cooperates with paxillin Pxl1, calcineurin phosphatase, F-BAR protein Cdc15, and Sbg1. This cooperation is essential for both contractile ring stability and septum formation.^32^^,^^33^^,^^34^^,^^35^^,^^36^ In this study, we unveil a non-conventional role for a β(1,3)glucan synthase and its β(1,3)glucan product in fission yeast growth polarity. We show that Bgs1-synthetized linear β(1,3)glucan cooperates specifically with the Tea1-Tea4 complex, but not with the rest of polarisome proteins, and all together are essential for the control and maintenance of growth polarity and morphology (Figure 5C). This cooperation suggests a redundant safety mechanism to ensure that the polarized growth will not be lost with just the failure of one of them. Similarly, Bgs1 linear β(1,3)glucan could be also ultimately responsible for the essential cooperation in the control of actomyosin ring stability and septum formation during cytokinesis. Furthermore, Bgs1-dependent β(1,3)glucan is necessary for the polarization of both actin patches and SRM domains, and this restricted localization of actin patches and SRM domains ultimately determinates the proper polarized growth. Remarkably, this shows the strong similarities in the essential functions of Bgs1 controlling actin structures in different processes, actomyosin ring during cytokinesis and actin patches during polarized growth. Bgs1 function in growth polarity is specific to this isoenzyme, as it does not occur with the other integral membrane glucan synthases, Bgs4 and Ags1, which are responsible for the synthesis of the major polysaccharides essential for cell wall integrity. The Bgs1-Tea1-Tea4-promoted rounded morphology differs from that observed in mutants of Bgs4 or Ags1 glucan synthases because: (i) Bgs4 or Ags1 mutants do not require the absence of Tea1 or Tea4 to induce spherical cells, (ii) the rounded morphology in Bgs4 or Ags1 mutants is caused by a strong cell wall synthesis defect, which is suppressed by sorbitol, (iii) Bgs1 depletion does not cause a strong cell wall defect, and (iv) Bgs1 depletion induces elongated but not rounded morphology.^22^^,^^23^^,^^24^^,^^25^^,^^28^^,^^75^^,^^76^

We further show that the Bgs1-mediated loss of growth polarity in the absence of the Tea1-Tea4 complex results from actin patches delocalization. This is evidenced by observing similar rounded morphology in cells only lacking the Tea1-Tea4 complex under conditions causing actin patches delocalization, such as (i) with mild LatB treatment, (ii) in the actin mutant *cps8-188*, or (iii) with defective actin patches lacking components like Myo1, Wsp1, or Arp3. Moreover, our study reveals that Bgs1 regulates the polarization of SRM domains and actin patches but not the opposite. We also show that Bgs1 physically interacts with End4 and Wsp1. In both fission and budding yeast, End4 and the WASP-Myo complex form part of two distinct endocytic modules based on function and timing of appearance. End4 is classified as a middle coat protein of the modules of early, middle, and late coat proteins (modules I and II), while Wsp1 and Myo1 are part of module III of WASP and myosin-related proteins, preceding module IV of actin and actin-associated proteins, and module V of scission-related proteins.^62^^,^^64^ Therefore, the physical interaction of Bgs1 with endocytic proteins is not specifically associated with a particular module. Although End4 and Wsp1 are found at the plasma membrane and endocytic vesicles,^63^^,^^64^ their interaction with Bgs1 likely occurs at the plasma membrane because: (1) Bgs1 is a large integral plasma membrane protein; and (2) in WT cells, actin patches are polarized and interact with Bgs1, whereas when Bgs1 is absent, they do not internalize but spread along the plasma membrane.

An important aspect uncovered in this study is the possible requirement of Bgs1 activity for its complete localization to the plasma membrane. Consequently, the non-functional but correctly localized GFP-Bgs1^9A(1136-1144)^ version requires the presence of the WT version of Bgs1 for its maximum localization to the plasma membrane. During Bgs1 depletion, the Bgs1^9A(1136-1144)^ localization is slightly decreased, as it is also observed with GFP-Bgs4 and Ags1-GFP during *bgs1*^*+*^ repression, in this and previous studies.^32^^,^^33^ This suggests that all the glucan synthases need the Bgs1 product of reaction, linear β(1,3)glucan, to stably remain in the plasma membrane. In any case, the slight GFP-Bgs1^9A(1136-1144)^ decrease during *bgs1*^+^ repression is not the cause of the emergence of morphological phenotype because much lower amounts of GFP-Bgs1 maintaining WT phenotype have been described in both P*nmt1*^*+*^-81-*GFP-bgs1*^+^ cells growing without thiamine (induced *GFP-bgs1*^*+*^), and *GFP-cps1-191* cells growing at permissive 25°C.^28^^,^^32^ In addition, the fact that the non-functional correctly localized Bgs1^9A(1136-1144)^ version fully remains in the membrane when an additional functional Bgs1 copy is present, but it slightly decreases during Bgs1 depletion, suggests a possible oligomerization in Bgs1 complexes, as previously proposed.^21^^,^^28^ This organization might be similar to the complexes of the structurally related cellulose synthase from plants, which forms hexameric complexes and the cellulose protofibers are extruded from a channel formed within individual monomers.^77^^,^^78^ In fact, cryo-electron microscopy studies of the structure of β(1,3)glucan synthase Fks1 from *S. cerevisiae* reveal strong similarities with cellulose synthase from bacteria and plants.^79^^,^^80^

As presented in this study, Bgs1 linear β(1,3)glucan is required for the polarization of both actin patches and SRM domains in *S pombe* (Figure 5C). As mentioned before, restriction of sterols to form SRM domains at the tips and septum depends on endocytosis, which is an event mediated by actin patches.^7^^,^^81^ The absence of Bgs1 linear β(1,3)glucan does not disrupt actin patches but leads to the delocalization of these F-actin structures throughout the cell periphery. Consequently, endocytosis is affected, and does not occur properly at focalized areas, leading to the dispersion of sterols all over the plasma membrane. This sterols spread is similar to what happens in cells with endocytosis defects, such as *myo1*Δ.^56^ Indeed, Bgs1-defective cells exhibit: (i) hypersensitivity to the Arp2/3 complex inhibitor CK-666; (ii) a significant delay in the internalization of the fluorescent dye FM4-64, suggesting that the actin patches are only partially functional; (iii) accumulation of smaller, heterogeneous vesicle-like structures, likely endosomes, distributed throughout the cytoplasm; and (iv) drastic fragmentation of the vacuoles. Altogether, these observations suggest an important role for Bgs1 in the control of membrane traffic from the plasma membrane to vacuoles. Sterols are known to be required for endocytosis in both animal and fungal cells. A previous study in *S. cerevisiae* showed that some mutants defective in ergosterol are able to uptake FM4-64, but exhibit defects in the FM4-64 movement from endosomal compartments to vacuoles.^82^ Interestingly, *S. cerevisiae* cells with mutations or deletion of the β(1,3)glucan synthase gene *Fks1* show reduced levels of Lucifer yellow uptake in the vacuole, suggesting that endocytosis is also defective in these mutants.^51^ Thus, it is possible that the role of Bgs1 in endocytosis could be a conserved function among fungi.

We also observed that Cpy1 sorting to the vacuole is malfunctioning in Bgs1-defective cells, providing additional evidence of membrane traffic defects in these cells. The F-actin formed by the active Arp2/3 complex is crucial for vacuole fusion,^83^ and SRM domains are required for the localization of proteins involved in membrane trafficking and fusion.^84^^,^^85^ Hence, the endocytosis and membrane traffic defects observed in Bgs1-defective cells may be caused by the dispersion of actin patches and absence of polarized SRM domains. In fact, the endocytosis and membrane traffic defects arise simultaneously with the actin patches delocalization and sterols spread in a time course (10 h of Bgs1 depletion), and long before the emergence of rounded or elongated and multiseptated morphological defects (15 h of Bgs1 depletion) (Figure S8D).

Extracellular Bgs1 linear β(1,3)glucan is essential for primary septum formation and cell separation during cytokinesis.^28^ Also, extracellular Bgs4 branched β(1,3)glucan is essential to connect the cell wall with the contractile ring during both ring maturation and septum ingression.^25^ The extracellular matrix is the functional equivalent of the cell wall in animal cells, and a similar function in cytokinesis has been suggested for some polymers of the extracellular matrix.^26^^,^^27^ The existence of the unique non-functional correctly localized GFP-Bgs1^9A(1136-1144)^ version allowed to discriminate between Bgs1 protein itself and its β(1,3)glucan product. This permitted us to uncover the essential role of extracellular linear β(1,3)glucan in the control of polarized growth, probably connecting to plasma membrane and actin patches to maintain actin patches and SRM domains polarization, as it was shown for the contractile ring during cytokinesis.^25^ Similarly, the extracellular matrix has been shown to be required for polarized growth and cell migration in neuronal cells.^38^^,^^39^^,^^40^ These and previous findings reveal strong functional similarities between the cell wall and the extracellular matrix of animal cells.

### Limitations of the study

The main limitations of this study are as follows: (i) This study is primarily based on genetic analysis and imaging-based methods to investigate the physiological effects of the absence of cell wall linear β(1,3)glucan and Bgs1. Additional *in vitro* biochemical methods should provide further evidence and insight into the molecular mechanism of the essential cooperation of linear β(1,3)glucan and Bgs1 with the Tea1-Tea4 complex. (ii) Bgs1 glucan synthase is a large integral plasma membrane protein, making it more challenging to study the molecular mechanisms of Bgs1 functions compared to the numerous possibilities available for studying soluble cytoplasmic proteins. Furthermore, Bgs1 is an essential protein, which makes it challenging to study significant alterations in its sequence, as these will result in cell death. Thus, this protein can only be studied using either thermosensitive mutants or *bgs1*^*+*^ gene repression strains. (iii) In contrast to the many techniques for labeling and detecting proteins, the number of methods for detecting and differentiating polysaccharides, such as the linear β(1,3)glucan, is very limited. Currently, the only available techniques that allow discrimination of the localization of linear and branched β(1,3)glucans are calcofluor staining for living cells and immunoelectron microscopy for fixed cells. In addition, the function of the linear β(1,3)glucan can only be indirectly inferred through examination of the effect caused by the absence or loss of function of Bgs1. (iv) Linear β(1,3)glucan cooperates with the Tea1-Tea4 complex. However, the molecular mechanism of this interaction is unknown. Similarly, the molecular mechanism of the interaction between actin patches and the Tea1-Tea4 complex remains to be elucidated. (v) Additionally, the absence of Bgs1 causes defects in endocytosis and membrane traffic. It would be interesting to explore whether these defects are due to the dispersion of actin patches and sterols throughout the membrane. (vi) Although it was not the subject of this study, linear β(1,3)glucan is also likely the ultimately responsible for the control of actomyosin ring stability and septum synthesis during cytokinesis. Further biochemical and structural analyses are required to determine how an extracellular polysaccharide, such as the linear β(1,3)glucan, might affect the proper homeostasis of the cytoplasmic actin cytoskeleton.

## STAR★Methods

### Key resources table

**Table:** 

REAGENT or RESOURCE	SOURCE	IDENTIFIER
**Antibodies**		

Mouse monoclonal anti-GFP clone JL-8	Takara Bio	632381; RRID: AB_10013427
Rabbit polyclonal anti-GFP	Thermo Fisher Scientific	A-6455; RRID: AB_221570
Mouse monoclonal anti-HA clone 12CA5	Roche	11666606001; RRID: AB_514506
Mouse monoclonal anti–α-tubulin clone B-5-1-2, ascites fluid	Sigma-Aldrich	T5168; RRID: AB_477579
Goat anti-mouse IgG-HRP conjugate	Bio-Rad	170–6516; RRID: AB_11125547
Mouse monoclonal anti-carboxypeptidase Y from *S. cerevisiae*, clone 10A5B5	Abcam/Thermo Fisher Scientific	ab113685; RRID: AB_10865711/A-6428; RRID: AB_2536203

**Experimental models: organisms/strains**		

Fission yeast strains used in this study are detailed in Table S2	This study	N/A

**Recombinant DNA**		

pJK148	Keeney and Boeke,^86^	https://doi.org/10.1093/genetics/136.3.849
pJK-*GFP-12A-bgs1*^*+*^	Ramos et al.^33^	https://doi.org/10.1083/jcb.201808163
pJK*-tdTom-12A-bgs1*^*+*^	Ramos et al.^33^	https://doi.org/10.1083/jcb.201808163
pJK-*GFP-12A-bgs4*^*+*^	Ramos et al.^33^	https://doi.org/10.1083/jcb.201808163
pJK-*ags1*^*+*^*(1–6267)-12A-GFP-12A*	Ramos et al.^33^	https://doi.org/10.1083/jcb.201808163
pJK-*GFP-12A-bgs1*^*9A(10-18)*^	This study	N/A
pJK-*GFP-12A-bgs1*^*9A(334-342)*^	This study	N/A
pJK-*GFP-12A-bgs1*^*9A(1136-1144)*^	This study	N/A
pAL-KS^+^	Ishiguro et al.^50^	https://doi.org/10.1128/jb.179.24.7653-7662.1997
pAL-*bgs4*^*+*^	Cortés et al.^21^	https://doi.org/10.1242/jcs.01585
pAL-*ags1*^*+*^	Cortés et al.^22^	https://doi.org/10.1083/jcb.201202015
p41XL	Moreno et al.^41^	https://doi.org/10.1002/1097-0061(20000630)16:9<861::AID-YEA577>3.0.CO;2-9
p41X- *bgs4*^*+*^	Muñoz et al.^25^	https://doi.org/10.1083/jcb.201304132
p41X-*ags1*^*+*^	Muñoz et al.^25^	https://doi.org/10.1083/jcb.201304132

**Software and algorithms**		

CW4000 cytoFISH	Leica Biosystems	https://www.leicabiosystems.com/
softWoRx 5.5.0	Applied Precision	https://www.appliedp.com/
MetaMorph 7.7.8	Molecular Devices	https://www.moleculardevices.com/
Fiji/ImageJ	National Institutes of Health	https://imagej.nih.gov/ij/
Adobe Photoshop CS2	Adobe	https://www.adobe.com/

**Other**		

μ-Slide 8 Well Uncoated	Ibidi	80821-uncoated
Protein A Sepharose CL-4B	GE Healthcare/Sigma-Aldrich	GE17-0780-01
Calcofluor white/Fluorescent Brightener 28	Sigma-Aldrich	F3543
Filipin complex from *Streptomyces filipinensis*	Sigma-Aldrich	F9765
Latrunculin B from *Latrunculia magnifica*	Sigma-Aldrich	L5288
Lectin from *Glycine max* (soybean)	Sigma-Aldrich	L1395
CK-666, Arp2/3 Complex Inhibitor I	Millipore Corp./Sigma-Aldrich	182515
FM4-64/SynaptoRed C2	Biotium	70021/70027
Thiamine hydrochloride	Sigma-Aldrich	T4625
Latrunculin A from *Latrunculia magnifica*	Enzo Life Sciences	BML-T119-0500
CellTracker Blue CMAC fluorescent dye	ABP Biosciences/Thermo Fisher Scientific	C037/11564237
CDCFDA, Carboxy-DCFDA (5-(and-6)-Carboxy-2′,7′-Dichlorofluorescein Diacetate), mixed isomers	Molecular Probes/Invitrogen/Thermo Fisher Scientific	C369/11560066
D-Sorbitol	Sigma-Aldrich	S1876

### Resource availability

#### Lead contact

Further information and requests for resources and reagents should be directed to and will be fulfilled by the lead contact. Juan C. Ribas (ribas@usal.es).

#### Materials availability

All yeast strains and plasmids used in this study will be made available upon request without any restriction.

#### Data and code availability

The data reported in this paper will be shared by the lead contact upon request.

This paper does not report original code.

Any additional information required to reanalyze the data reported in this paper is available from the lead contact upon request.

### Experimental model and study participant details

#### Strains and culture conditions

The *S. pombe* strains used in this study are detailed in Table S2 (see key resources table). General procedures for yeast and bacterial culture, as well as genetic manipulations, were performed according to previously established protocols.^87^^,^^88^ Strains were made through genetic crosses employing either tetrad dissection or random spore selection against the corresponding parental auxotrophies or antibiotic resistances. Early log-phase cells were cultured at different temperatures (ranging from 25°C to 36°C) in either rich medium (YES) or Edinburgh minimal medium (MM) with appropriate supplements, and without or with 1.3 M sorbitol (- S, + S) as an osmotic stabilizer. For repression experiments in strains carrying the thiamine-repressible *nmt1*^*+*^-81 promoter,^41^ early log-phase cells cultivated in MM without or with sorbitol were diluted with the same medium plus thiamine (+ T) at a final concentration of 20 μg/ml. Sporulation was induced on solid sporulation agar SPA medium. The actin-depolymerizing and monomer-sequestering drug Latrunculin A (LatA, Enzo Life Sciences) was applied at a final concentration of 100 μM (from a 10 mM stock in DMSO). Under these conditions of high excess of LatA, all F-actin structures of cables, patches, and the contractile ring, were depolymerized in less than 5 min. Latrunculin B (LatB, Sigma-Aldrich) was used at a final concentration of 20 μM (from a 10 mM stock in DMSO). Under these conditions of low concentration of LatB, actin patches were delocalized but not depolymerized. Lower concentrations of LatB did not affect the localization of actin patches, while higher concentrations caused the depolymerization of actin patches.

### Method details

#### Construction of mutant and tagged strains

The *bgs1*Δ*::ura4*^*+*^ P*nmt1*^*+*^-81-*bgs1*^+^ strain 435 has been previously described^28^ and contains the *bgs1*Δ*::ura4*^*+*^ deletion and a single integrated *bgs1*^+^ copy expressed under the control of the P*nmt1*^*+*^-81 version (very low expression) of the thiamine-repressible *nmt1*^+^ promoter.^41^ The P*nmt1*^*+*^-81-*bgs1*^+^ strain 1506 contains the *bgs1*^*+*^ promoter replaced by a sequence containing the selection marker *ura4*^*+*^ adjacent to the *nmt1*^*+*^-81 promoter, followed by the *bgs1*^+^ coding sequence. This strain was obtained from a WT diploid strain through homologous recombination of a PCR-amplified cassette containing the sequence 5′UTR*bgs1*^+^-*ura4*^*+*^-P*nmt1*^*+*^-81-5′*bgs1*^+^ (1.0 kb of 5′*bgs1*^*+*^ ORF) from pSK-5′UTR*bgs1*^+^-*ura4*^*+*^-P*nmt1*^*+*^-81-*bgs1*^+^ and subsequent sporulation. The haploid P*nmt1*^*+*^-81-*bgs1*^+^ strain lacks the native *bgs1*^*+*^ promoter and contains a single integrated *bgs1*^+^ copy expressed under the control of the *nmt1*^*+*^-81 promoter. Other P*nmt1*^*+*^-81-*bgs1*^+^ strains were made from strain 1506 through random spore selection against auxotrophies or selection markers of both parental strains. All P*nmt1*^*+*^-81-*bgs1*^+^ strains exhibited the phenotype of elongated, branched, and multiseptated cells in the presence of thiamine (+ T, repressed conditions) and a WT phenotype in its absence (- T, induced conditions), as previously described.^28^^,^^32^ *GFP-bgs1*^+^, *GFP-bgs4*^+^, *ags1*^*+*^*-GFP,* P*nmt1*^*+*^-81-*bgs4*^+^, and P*nmt1*^*+*^-81-*ags1*^+^ strains were made as described.^22^^,^^25^^,^^32^ The strains P*nmt1*^*+*^-81-*bgs4*^+^ and P*nmt1*^*+*^-81-*ags1*^+^ exhibited a strong lytic phenotype in the presence of thiamine and a WT phenotype in its absence, as reported.^22^^,^^25^

The original *cps1-191* mutant strain of *bgs1*^*+*^ has recently been reported to carry an additional mutation in the *mto2*^*+*^ gene sequence, resulting in the S338N amino acid change.^54^ To ascertain whether the *cps1-191* strains used in this study harbor the described Mto2-S338N substitution, the *mto2*^*+*^ gene of the original *cps1-191* strain 972, its *cps1-191* descendant strains 1024 and 1025, and their subsequent *cps1-191* descendant strains 6824, PPG14753, PPG14755, PPG14759, PPG15349, and PPG14769, was sequenced and analyzed. The remaining *cps1-191* strains are transformants from 1025, 6824, and PPG14755, which have already undergone analysis. Only the original strain 972 exhibited the Mto2-S338N substitution (nucleotide change AGT to AAT). In contrast, the descendant strains 1024 and 1025, and hence their progeny, contained the WT *mto2*^*+*^ sequence.

Strains 2339, 3477, 3899, and 3950 containing the WT, 9A functional, and 9A non-functional versions of *bgs1*^*+*^, were generated by transformation of the P*nmt1*^*+*^-81-*bgs1*^+^ strain 2100 with WT pJK-*GFP-12A-bgs1*^*+*^, functional mutant pJK-*GFP-12A-bgs1*^*9A(10-18)*^, non-functional non-localized mutant pJK-*GFP-12A-bgs1*^*9A(334-342)*^, and non-functional correctly localized mutant pJK-*GFP-12A-bgs1*^*9A(1136-1144)*^, respectively, previously cut with Eco47III to direct their integration into the *leu1-32* locus of the genome (Figure S4). Addition of thiamine to the culture medium repressed the WT *bgs1*^*+*^ copy under the control of the P*nmt1*^*+*^-81 promoter, maintaining the cells with the sole expression of the additional copy of WT pJK-*GFP-12A-bgs1*^*+*^, functional mutant pJK-*GFP-12A-bgs1*^*9A(10-18)*^, non-functional non-localized mutant pJK-*GFP-12A-bgs1*^*9A(334-342)*^, and non-functional correctly localized mutant pJK-*GFP-12A-bgs1*^*9A(1136-1144)*^, respectively. Strains 4464, 4407, and 4530, containing the non-functional *bgs1*^*9A(1136-1144)*^ version of *bgs1*^+^, were constructed by transforming the P*nmt1*^*+*^-81-*bgs1*^+^ strain 2076, P*nmt1*^*+*^-81-*bgs1*^+^ *tea1*Δ strain 2507, and P*nmt1*^*+*^-81-*bgs1*^+^ *crn1*^*+*^*-GFP* strain 2886, with pJK-*GFP-12A-bgs1*^*9A(1136-1144)*^ or pJK-*tdTom-12A-bgs1*^*9A(1136-1144)*^, cut with Eco47III for integration into the *leu1-32* locus. Thiamine addition to the culture medium repressed the WT *bgs1*^*+*^ copy, maintaining the expression of only the non-functional *bgs1*^*9A(1136-1144)*^ mutant copy.

Plasmids pJK-*GFP-12A-bgs1*^*+*^, pJK*-tdTom-12A-bgs1*^*+*^, pJK-*GFP-12A-bgs4*^*+*^, and pJK-*ags1*^*+*^*(1-6267)-12A-GFP-12A* have been described elsewhere.^33^ These plasmids are the integrative plasmid pJK148 (*leu1*^+^ selection) with a 9.6-kb *GFP-12A-bgs1*^*+*^, 10.2-kb *tdTom-12A-bgs1*^*+*^, 9.6-kb *GFP-12A-bgs4*^*+*^, and 9.9-kb *ags1*^*+*^*(1-6267)-12A-GFP-12A*, respectively. Plasmid pJK-*ags1*^*+*^*(1-6267)-12A-GFP-12A* contains *12A-GFP-12A* inserted in-frame at base 5866 (amino acid 1956) of the *ags1*^*+*^ coding sequence. Plasmids pJK-*GFP-12A-bgs1*^*9A(10-18)*^, pJK-*GFP-12A-bgs1*^*9A(334-342)*^, and pJK-*GFP-12A-bgs1*^*9A(1136-1144)*^ are derivatives of pJK-*GFP-12A-bgs1*^*+*^ with 9A substitutions at amino acids 10-18, 334-342, and 1136-1144 of hydrophilic regions of the *bgs1*^*+*^ ORF, respectively.

Plasmids pAL-*bgs4*^*+*^ and pAL-*ags1*^*+*^ have been described previously.^21^^,^^22^ These plasmids are the multicopy plasmid pAL-KS^+^ (*S. cerevisiae LEU2* selection) with an 8.8-kb *bgs4*^*+*^ fragment and an 11.2-kb *ags1*^*+*^ fragment, respectively. These multicopy plasmids contain the corresponding ORF and its native promoter sequence, and were used for overexpression studies.

Plasmids p41X-*bgs4*^*+*^ and p41X-*ags1*^*+*^ have been described elsewhere.^25^ These plasmids are the multicopy P*nmt1*^*+*^-41-containing thiamine-repressible plasmid pJR-41XL^41^ with the 5.9-kb *bgs4*^*+*^ ORF and 7.2-kb *ags1*^*+*^ ORF sequence, respectively. Induced expression levels of the P*nmt1*^*+*^-41 promoter are 10- to 50-fold higher than those of the P*nmt1*^*+*^-81 version and 5- to 20-fold lower than those of the P*nmt1*^*+*^-3 version. Higher overexpression of these ORFs with the P*nmt1*^*+*^-3 version is lethal for the cell. Therefore, the P*nmt1*^*+*^-41 version was selected for overexpression studies at induced expression levels.

#### Recombinant DNA methods

All DNA manipulations were performed through established methods. Enzymes were used according to the recommendations of the suppliers. Transformation of *S. pombe* was carried out using the lithium acetate method.^89^

#### Immunoprecipitation and immunoblot analysis

Early log-phase cells (10^9^ cells in 100 ml) expressing the different tagged proteins were harvested (1,500 g, 5 min, 4°C), washed once with cold STOP solution (154 mM NaCl, 10 mM EDTA, 10mM NaN_3_, 10mM NaF) and once with cold WASH buffer (50mM Tris-HCl, pH 7.5, 5 mM EDTA). Cell lysis was performed by resuspending cells in 200 μl of lysis buffer (50 mM Tris-HCl, pH 7.5, 1 mM EDTA, 200 mM NaCl, 5 mM phenylmethylsulphonylfluoride, and 2 μg/ml aprotinin, leupeptin, and pepstatin) and breaking them with glass beads (FastPrep FP120, 2 x 15 s and 1 x 20 s, speed of 5.5; MP Biomedicals; Thermo Fisher Scientific). The total cell extracts were collected, centrifuged (21,000 g, 5 min, 4°C), and homogenized again in the same supernatant. This last step improved the disaggregation and immunodetection of membrane proteins from total cell extracts. Cell debris was removed by centrifugation (4,500 g, 1 min, 4°C). The supernatant was collected and then, for cell extracts analysis, urea was added to a final concentration of 0.5 M, and the extracts were agitated for 1 h in a Thermomixer (1,300 rpm, 1°C; Thermo Fisher Scientific). Finally, samples were diluted with concentrated sample buffer to reach a 1X concentration (1X is 50 mM Tris-HCl, pH 6.8, 2% SDS, 1% 2-mercaptoethanol, 12.5 mM EDTA, 10% glycerol, and 0.02% bromophenol blue) and stored at -80°C. Protein concentration was determined using the Bradford assay.

For immunoprecipitation analysis, the supernatant was centrifuged (21,000 g, 30 min, 4°C), and the membranes pellet was resuspended in 200 μl of immunoprecipitation buffer (IPB; 50 mM Tris-HCl, pH 7.5, 5 mM EDTA, 200 mM NaCl, 0.5% Tween 20, 100 μM phenylmethylsulphonylfluoride, and 2 μg/ml leupeptin and aprotinin). The membrane suspension was agitated for 30 min in a Thermomixer (1,300 rpm, 1°C; Thermo Fisher Scientific), centrifuged (21,000 *g*, 30 min, 4°C), and the supernatant was collected and diluted with IPB. The solubilized membrane proteins (2 mg total protein) were incubated with rabbit polyclonal anti-GFP serum (Thermo Fisher Scientific, Molecular Probes, Invitrogen Cat. no. A-6455) and protein A-sepharose beads (Cat. no. 17-0780-01; GE Healthcare) for 4 h at 4°C, washed three times with IPB and boiled in sample buffer.

Proteins were separated in 3-8% Tris-Acetate SDS–PAGE (NuPAGE; Invitrogen), transferred to Immobilon-P membranes (EMD Millipore), and blotted using mouse monoclonal anti-GFP (1:2,000; JL-8, Takara Bio Cat no. 632381), mouse monoclonal anti-HA (1:10,000, 12CA5, Roche Diagnostics Cat no. 11666606001), or mouse monoclonal anti–α-tubulin (1:10,000; B-5-1-2, Sigma-Aldrich Cat no. T5168) antibodies. Immunodetection was performed with goat anti–mouse IgG-horseradish peroxidase conjugate antibody (1:10,000; Bio-Rad Laboratories Cat. no. 170-6516) and the ECL Plus detection kit (GE Healthcare). Western blot analysis of cell extracts (80 μg total protein) was performed to determine the total amount of tagged proteins. Cell extracts and western blots were repeated in at least three independent experiments.

#### Microscopy techniques and image analysis

For cell wall staining, early log-phase cells were cultivated at the corresponding temperature in liquid media (YES or MM) without or with sorbitol, and either in the absence or presence of 20 μg/ml thiamine for cells expressing or repressing *bgs1*^+^, respectively. Before imaging, cells were concentrated (1,000 g, 1 min) and resuspended in a low volume (20-50 μl) of the culture medium. The cell wall was visualized by adding a solution of the fluorochrome Calcofluor White (CW; 50 μg/ml final concentration, from a 10 mg/ml stock solution; Fluorescent Brightener 28, Sigma-Aldrich) to the sample and using the appropriate filter. Cells with GFP- or RFP-labelled proteins were directly visualized using the appropriate filters. For sterols staining, 5 μl of a fresh solution of 50 μg/ml filipin (from a stock solution of 1 mg/ml in DMSO; Sigma-Aldrich Cat no. F9765) was added to the 50-μl cell sample (5 μg/ml final concentration). After incubation for 1-2 min at room temperature in the dark, the cells were visualized using the excitation light of 360 nm and the appropriate filter. Alexa Fluor 448-conjugated phalloidin staining to visualize the F-actin was performed with early log-phase cells fixed for 40-60 min with 1/4 volume of paraformaldehyde (16% EM-grade MeOH-Free, Polysciences) and 1/10 volume of PEM buffer (10 mM EGTA; 1 mM MgCl_2_; 100 mM PIPES, pH 6.8). The cells were then washed 3x with PEM, resuspended in PEM with 1% Triton X-100 for 30 seconds, and washed 3x with PEM. Subsequently, cells (2ul) were stained with 8 μl of 5 mg/ml Alexa Fluor 488-conjugated phalloidin (Thermo Fisher Scientific) and incubated for 1h at room temperature or overnight at 4ºC rocking in the dark.

To examine endocytosis from the plasma membrane, 2 ml of early log-phase cells growing in MM + S were centrifuged and washed three times with cold medium (3,500 g, 1 min), resuspended in the same medium, and kept on ice for 5 min. The fluorescent dye FM4-64 ^48^^,^^69^ was then added to the cells at a final concentration of 40 μM (from a stock solution of 10 mM in water), followed by immediate examination through time-course FM4-64 fluorescence microscopy at the indicated times with FM4-64 (0, 3, 6, 9, 12, 15 and 18 min) and using the appropriate filter. The kinetics of transport to vacuoles were examined by adding FM4-64 to the cells in MM or MM + T at a final concentration of 80 μM. Cells were incubated with FM4-64 on ice in the dark for 30 min and washed with cold medium before being observed (time 0). Subsequently, washed cells were incubated for the indicated times (0, 15, 30, 45 and 60 min) at 30°C (repressed P*nmt1*^*+*^-81-*bgs1*^+^ cells) or 34ºC (*cps1-191* mutant cells) before being imaged using time-course microscopy.

Vacuole staining of *S. pombe* cells with the fluorescein derivative 5-(and 6-carboxy 2′,7′-dichlorohydrofluorescein diacetate, bis(acetoxymethyl) ester (CDCFDA) was performed essentially as described.^90^ Early log-phase P*nmt1*^*+*^-81-*bgs1*^+^ cells grown at 28°C in MM (*bgs1*^*+*^-induced) and transferred to MM with 20 μg/ml thiamine (+ T, *bgs1*^*+*^-repressed) for 15 h (Figure 7A), and *cps1-191* mutant cells grown in MM at permissive 25°C and restrictive 34°C for 4 h (Figure 7B), were collected by centrifugation and resuspended in YES medium (1 ml) containing 10 mM citric acid. The cells were centrifuged again, resuspended in 1 ml of YES with 10mM citric acid and 25 μM CDCFDA (from a 10 mM stock in DMSO; Molecular Probes Cat no. C369) and incubated at 30°C for 30 min in the dark. Finally, the cells were washed three times with 1 m of YES with 10mM citric acid and resuspended in a small volume of the same medium for microscopy observation. CDCFDA fluorescence was detected upon sample excitation at 488 nm.

To visualize the vacuolar lumen (Figure S8C), CellTrack Blue CMAC (ABP Biosciences Cat no. C037) was used.^70^ Early log-phase P*nmt1*^*+*^-81-*bgs1*^+^ cells grown at 28°C in MM and transferred to MM + T were collected after 5, 10, 15, and 24 h of *bgs1*^*+*^ repression with thiamine, and resuspended in 0.5 ml of MM with 100 μM Blue CMAC (from a 10mM stock in DMSO). The cells were then incubated at room temperature for 30 min in the dark, washed with 1ml of MM by shaking for 5 min, and collected for time-course microscopy observation. Blue CMAC fluorescence was detected upon sample excitation at 405 nm.

Images were obtained using various microscopy setups. A Leica DM RXA fluorescence microscope equipped with PL APO 63×/1.32 oil PH3 and PL FLUOTAR 100x/1.30 oil PH3 objectives, a digital camera (DFC350FX; Leica Biosystems) and CW4000 cytoFISH software (Leica Biosystems) was used. Differential interference contrast (DIC, Figures 3A and S8C) images were obtained with a Nikon eclipse 90i microscope equipped with a PlanApo 60x/1.40 oil objective, an ORCA ER camera (Hamamatsu), and MetaMorph 7.7.8 software (Molecular Devices). Other images were obtained with an Olympus IX71 fluorescence microscope equipped with a PlanApo 100x/1.40 IX70 objective, a Personal DeltaVision system, a Solid-State Illumination System InsightSSI™ Spectra7 (Applied Precision), a CoolSnap HQ2 monochrome camera (Photometrics), and softWoRx 5.5.0 imaging software (Applied Precision). Images were processed with Fiji / Image J (National Institutes of Health) and Adobe Photoshop CS2 software. All the analyses were repeated in three to four independent experiments, and representative images of the analyzed phenotypes were interchangeably selected from the experiments.

Time-lapse video imaging was performed essentially as described.^22^ 0.5 ml of early log-phase cells grown in MM at 28°C and transferred to MM + T for 15 h were collected by low-speed centrifugation (2,000 g for 30 s), resuspended in 300 μl of the same medium, and placed in a well from a μ-Slide 8-well (Ibidi Cat no. 80821-Uncoated) previously coated with 10 μl of 1 mg/ml soybean lectin (Sigma-Aldrich Cat no. L1395). Time-lapse video experiments were made at 28°C by acquiring epifluorescence cell images in single planes and 1 × 1 binning on an inverted microscope (model IX71; Olympus) equipped with a PlanApo 100×/1.40 IX70 objective and a Personal DeltaVision system (Applied Precision). Images were captured using a CoolSnap HQ2 monochrome camera (Photometrics) and softWoRx 5.5.0 imaging software (Applied Precision) and restored by 3D Deconvolution (conservative ratio, 10 iterations, and high noise filtering) through softWoRx imaging software. Subsequently, images were processed (color, brightness, and/or contrast) with Fiji / ImageJ and Adobe Photoshop CS2 software. The time-lapse videos were repeated in three independent experiments.

For maximal intensity projections of WT and *bgs1*^*+*^-repressed cells, images were acquired in Z-stacks consisting of 31 sections spaced at 0.4 μm intervals to ensure complete coverage of the entire cell. The images were subjected to 3-D deconvolution using the softWorRx imaging software. Subsequently, only the sections that captured the cells (10 sections at 0 h, 18 at 24 h, and 21 at 36 h) were processed using the stacks and 3D projection functions in Fiji / Image J software.

#### Transmission electron microscopy

Early logarithmic phase cells were fixed with 2% glutaraldehyde EM (GA; Electron Microscopy Science) in 50 mM phosphate buffer, pH 7.2, 150 mM NaCl (PBS) for 2 h at 4°C, post-fixed with 1.2% potassium permanganate overnight at 4°C, and embedded in Quetol 812 as described.^91^ Ultrathin sections were stained in 4% uranyl acetate and 0.4% lead citrate and viewed with a transmission electron microscope (model H-800; Hitachi) operating at 125 kV.

#### Other methods

Plate assays to assess growth sensitivity to different temperatures or compounds were conducted by spotting 5 μl of appropriate 1/4 dilutions of early log-phase cells (10^7^ cells, A_600_ of 1.0) on YES solid medium containing 2% (w/v) bacto-agar supplemented with the corresponding compounds. Plates were incubated for 3 days at the specified temperatures. All assays were repeated in three independent experiments. The stock of the Arp2/3 complex inhibitor CK-666 (Sigma-Aldrich) was prepared at 10 mM concentration in DMSO.

Colony immunoblot analysis for the detection of secreted Cpy1 was performed as described^92^ with some modifications. Exponentially growing cells (2 × 10^7^ cells, A_600_ of 2.0) were spotted (5 μl) onto nitrocellulose membranes placed on YES agar plates and incubated at 25 and 32ºC for 24 h. After cell removal by washing, nitrocellulose membranes were subjected to immunodetection of Cpy1 using a mouse monoclonal antibody against Carboxypeptidase Y from *S. cerevisiae* (1:125; 10A5B5, Abcam Cat no. ab113685, Thermo Fisher Scientific Cat no. A-6428). Densitometry of colony blots was performed using the Fiji image processing package.^93^ Mean density units relative to WT were calculated from three independent experiments and plotted as relative mean density values ± SD.

### Quantification and statistical analysis

The number of samples analyzed (n) is defined in each figure and were derived from at least three independent experiments. The error bars correspond to standard deviation (SD) between experiments and are specifically indicated in each figure. Statistical analyses were done using Graphpad Prism software using the tests specified in the figure legends. Comparisons of two conditions were tested by unpaired t test. Statistical significances were marked by ∗p < 0.05, ∗∗p < 0.01, ∗∗∗p < 0.001, ∗∗∗∗p < 0.0001, and n. s.: not significant (p > 0.05).

## Supplemental information

Document S1. Figures S1–S8 and Table S1

Table S2. Fission yeast strains used in this study, related to STAR Methods
