# Supplementary material for: Fission yeast Bgs1 glucan synthase participates in the control of growth polarity and membrane traffic
Source: iScience. 2024 Jul 8;27(8):110477. doi: 10.1016/j.isci.2024.110477 (PMC11326927; doi:10.1016/j.isci.2024.110477)
Supplement: Document S1. Figures S1–S8 and Table S1 [file mmc1.pdf]

iScience, Volume *27*

Supplemental information

Fission yeast Bgs1 glucan synthase participates in the control of growth polarity and membrane traffic

Mariona Ramos, Rebeca Martín-García, M. Ángeles Curto, Laura Gómez-Delgado, M. Belén Moreno, Mamiko Sato, Elvira Portales, Masako Osumi, Sergio A. Rincón, Pilar Pérez, Juan C. Ribas, and Juan C.G. Cortés

SUPPLEMENTAL FIGURE LEGENDS

Figure S1.

Mod5, Bud6, For3, Cdc42, Shk1, Shk2, or Tea3 do not cooperate with Bgs1 in the control and maintenance of growth polarity. The simultaneous absence of multiple polarity factors is unable to induce a growth polarity loss similar to that elicited with the joint absence of Bgs1 and the specific Tea1-Tea4 complex.

Related to Figure 1, and Tables 1 and S1.

(A) Time-course fluorescence micrographs of calcofluor white (CW)-stained cells carrying *bgs1*$^{+}$ under the control of the thiamine repressible *nmt1*$^{+}$-81 promoter (P*nmt1*$^{+}$-81-*bgs1*$^{+}$). Cells were grown to early log-phase at 28ºC in Minimal Medium with 1.3 M sorbitol (MM + S; *bgs1*$^{+}$ ON, - T), transferred to MM + S with thiamine (MM + T + S; *bgs1*$^{+}$ OFF, +T), and imaged for CW fluorescence at the indicated times of *bgs1*$^{+}$ repression with thiamine (+T; 0, 24, 36, 48, 60, and 72 h). Arrow, bifurcated tips with two areas of cell growth.

(B) Fluorescence micrographs of CW-stained *mod5*Δ, *bud6*Δ, *for3*Δ, *tea3*Δ, *cdc42-D76G*, *cdc42-L160S*, *shk1-34* (*orb2-34*), and *shk2*Δ cells carrying P*nmt1*$^{+}$-81-*bgs1*$^{+}$. Cells were grown to early log-phase at 28ºC in Minimal Medium (MM; *bgs1*$^{+}$ ON, - T), transferred to MM with thiamine (MM + T; *bgs1*$^{+}$ OFF, +T), and imaged for CW fluorescence at the indicated times of *bgs1*$^{+}$ repression with thiamine (0 and 24 h).

(C) Triple deletion mutants of polarity factors *tea1*Δ, *tea4*Δ, *mod5*Δ, *bud6*Δ, and *for3*Δ were grown to early log-phase at 28ºC in YES medium and imaged for phase contrast and CW fluorescence. Scale bars, 5 µm.

Figure S2.

The glucan synthases Bgs1, Bgs4, and Ags1 remain correctly localized to the growing sites in the absence of Tea1 or Tea4. Similarly, Tea1, Tea4, Bgs4, and Ags1 stay correctly polarized in the absence of Bgs1.

Related to Figures 2 and S3.

(A) Fluorescence micrographs of CW-stained *tea1*Δ (left panels, first to third panels) and *tea4*Δ (right panels, fourth to sixth panels) cells carrying either *GFP-bgs1*$^{+}$, *GFP-bgs4*$^{+}$, or *ags1*$^{+}$*-GFP*. Cells were

grown to early log-phase at 28ºC in MM and imaged for CW and GFP fluorescence microscopy. Arrow: GFP-Bgs1, GFP-Bgs4, and Ags1-GFP localizations to the ectopic growing pole of *tea1*Δ and *tea4*Δ cells.

(B) Time-course fluorescence micrographs of CW-stained P*nmt1*$^{+}$-81-*bgs1*$^{+}$ cells carrying either *tea1*$^{+}$-*GFP*, *tea4*$^{+}$-*GFP*, *GFP-bgs4*$^{+}$, or *ags1*$^{+}$-*GFP*. Cells were grown to early log-phase at 28ºC in MM + S, transferred to MM + T + S, and imaged for CW and GFP fluorescences at the indicated times of *bgs1*$^{+}$ repression with thiamine (0, 15, and 24 h). Scale bars, 5 µm.

Figure S3.

The simultaneous absence of Bgs1 and Tea1-Tea4 complex induces the spreading of the other glucan synthases Bgs4 and Ags1 around the growing spherical cell. Bgs4 and Ags1, however, do not participate in the collaboration with the Tea1-Tea4 complex to regulate and maintain growth polarity.

Related to Figures 2, 3, and S2.

(A) Time-course fluorescence micrographs of CW-stained P*nmt1*$^{+}$-81-*bgs1*$^{+}$ (left panels) and *tea4*Δ P*nmt1*$^{+}$-81-*bgs1*$^{+}$ (right panels) cells carrying either *GFP-bgs4*$^{+}$ or *ags1*$^{+}$-*GFP*. Cells were grown to early log-phase at 28ºC in MM, transferred to MM + T, and imaged for CW and GFP fluorescences at the indicated times with thiamine (0, 15, and 24 h).

(B) Time-lapse sequences of *tea1*Δ P*nmt1*$^{+}$-81-*bgs1*$^{+}$ cells carrying *ags1*$^{+}$-*GFP*, illustrating the onset of formation of spherical cells with spreading of Ags1-GFP (cells 1, 2, and 3), to create enlarged rounded cells while maintaining Ags1-GFP around the cell. Early log-phase *ags1*$^{+}$-*GFP tea1*Δ P*nmt1*$^{+}$-81-*bgs1*$^{+}$ cells grown at 28ºC in MM were transferred to MM + T for 15 h and imaged for 5 h through GFP fluorescence time-lapse video microscopy. Elapsed time is shown in hours and minutes.

(C) Fluorescence micrographs of CW-stained P*nmt1*$^{+}$-81-*bgs4*$^{+}$ (left panels, first and second columns) and P*nmt1*$^{+}$-81-*ags1*$^{+}$ (right panels, third and fourth columns) cells either in the presence or absence of *tea4*$^{+}$ (*tea4*$^{+}$, first and third columns; *tea4*Δ, second and fourth columns). Cells were grown to early log-phase at 28ºC in MM + S, transferred to MM + T + S, and imaged for CW fluorescence at the indicated times of *bgs4*$^{+}$ or *ags1*$^{+}$ repression with thiamine. The selected repression times of *bgs4*$^{+}$ and *ags1*$^{+}$ were the maximum time of repression when cell lysis started to be detected in each case (9 and 7 h,

respectively). The start of cell lysis was considered the glucan synthase repression limit for morphological analysis in the absence of the corresponding glucan synthase.

(D) Phase contrast micrographs depicting the morphology of P*nmt1*$^{+}$-81-*bgs1*$^{+}$ (left panels, first to third panels), *tea1*Δ P*nmt1*$^{+}$-81-*bgs1*$^{+}$ (middle panels, fourth to sixth panels), and *tea4*Δ P*nmt1*$^{+}$-81-*bgs1*$^{+}$ (right panels, seventh to ninth panels) cells transformed with the multicopy plasmid pAL-KS^{+}, either empty (negative control, first, fourth and seventh panels), or overexpressing *bgs4*$^{+}$ (*bgs4*$^{+}$ OE; second, fifth and eighth panels) or *ags1*$^{+}$ (*ags1*$^{+}$ OE; third, sixth, and ninth panels). Early log-phase cells were grown at 28ºC in MM, transferred to MM + T for 24 h (*bgs1*$^{+}$ OFF, +T), and imaged for phase contrast microscopy.

(E) Phase contrast micrographs depicting the morphology of *cps1-191* (left panels, first to third panels), *tea1*Δ *cps1-191* (middle panels, fourth to sixth panels), and *tea4*Δ *cps1-191* (right panels, seventh to ninth panels) cells transformed with the overexpression plasmid p41X, either empty (negative control, first, fourth and seventh panels), or overexpressing *bgs4*$^{+}$ (*bgs4*$^{+}$ OE; second, fifth and eighth panels) or *ags1*$^{+}$ (*ags1*$^{+}$ OE; panels third, sixth, and ninth panels). Early log-phase cells were grown at 25ºC in YES + T medium, transferred to MM in the absence of thiamine at 30ºC for 24 h (-T; *bgs4*$^{+}$ OE, or *ags1*$^{+}$ OE), and imaged for phase contrast microscopy. *cps1-191* is a thermosensitive mutant allele of *bgs1*$^{+}$. Scale bars, 5 µm.

Figure S4.

A specific 9-alanine substitution generates a unique non-functional correctly localized GFP-Bgs1^{9A} mutant version.

Related to Figures 3 and S5.

Time-course fluorescence micrographs of CW-stained P*nmt1*$^{+}$-81-*bgs1*$^{+}$ cells:

(A) Without an additional *GFP-bgs1*$^{+}$ copy, serving as a control for the phenotype of *bgs1*$^{+}$ repression with thiamine.

(B) Carrying an additional wild-type *GFP-bgs1*$^{+}$ copy, serving as a control for GFP-Bgs1 localization and the absence of the *bgs1*$^{+}$ repression phenotype with thiamine.

(C) Carrying an additional functional *GFP-bgs1*$^{9A(10\text{-}18)}$ mutant copy, serving as an example of correct GFP-Bgs1 localization and the absence of the *bgs1*$^{+}$ repression phenotype with thiamine.

(D) Carrying an additional non-functional non-localized *GFP-bgs1*$^{9A(334\text{-}342)}$ mutant copy, serving as an example of the typical absence of GFP-Bgs1 localization in the cells with wild-type phenotype in the absence of thiamine (induced P*nmt1*$^{+}$-81-*bgs1*$^{+}$) and during the generation of the *bgs1*$^{+}$ repression phenotype with thiamine (repressed P*nmt1*$^{+}$-81-*bgs1*$^{+}$).

(E) Additionally carrying the non-functional correctly localized *GFP-bgs1*$^{9A(1136\text{-}1144)}$ mutant copy, displaying correct GFP-Bgs1 localization to poles and septum in the cells with wild-type phenotype in the absence of thiamine and also maintaining its correct localization to poles and septum while simultaneously generating the *bgs1*$^{+}$ repression phenotype with thiamine (repressed P*nmt1*$^{+}$-81-*bgs1*$^{+}$). This indicates that this correctly localized copy of GFP-Bgs1^{9A} is unable to maintain the wild-type cell state when the P*nmt1*$^{+}$-81-*bgs1*$^{+}$ copy is repressed. P*nmt1*$^{+}$-81-*bgs1*$^{+}$ cells in A to E were cultured to early log-phase at 28ºC in MM + S, transferred to MM + T + S, and imaged for CW and GFP fluorescences using time-course microscopy at the indicated times with thiamine (0, 5, 10, 15, and 24 h). Scale bars, 5 µm.

Figure S5.

The sole absence of Bgs1 function, with the GFP-Bgs1 mutant protein remaining correctly localized, leads to the diffusion of SRM domains and delocalization of actin patches throughout the plasma membrane.

Related to Figures 4, 5, S4 and S6.

(A) Fluorescence micrographs of P*nmt1*$^{+}$-81-*bgs1*$^{+}$ cells carrying *Cherry-D4H* and *crn1*$^{+}$*-GFP*. Cells were cultured to early log-phase at 28ºC in MM, transferred to MM + T, and imaged for Cherry-D4H and coronin Crn1-GFP fluorescences using time-course microscopy at the specified times with thiamine (0, 5, 10, 15, and 24 h).

(B) Fluorescence micrographs of P*nmt1*$^{+}$-81-*bgs1*$^{+}$ cells carrying either *end4*$^{+}$*-GFP* or *wsp1*$^{+}$*-YFP* to visualize actin patches. Cells were grown to early log-phase at 28ºC in MM + S, transferred to MM + T + S, and imaged for GFP or YFP fluorescence using time-course microscopy at the specified times with thiamine (0, 24, and 36 h).

(C) P*nmt1*$^{+}$-81-*bgs1*$^{+}$ cells grown at 28ºC in MM + S either in the absence or presence of thiamine, were stained with Alexa Fluor 488-phalloidin to observe actin patches. Images were captured for Alexa Fluor

488 fluorescence using time-course microscopy at the specified times with thiamine (0, 24, and 36 h). The images of actin patches in the left panels are a single section of the Z-stack. The images in the right panels are maximal intensity projections of 31 Z-sections spaced at 0.4 µm intervals, selecting only the sections that captured the cells (10 in 0 h, 18 in 24 h, and 21 in 36 h).

(D) Fluorescence micrographs of filipin-stained WT and *cps1-191* mutant cells carrying Lifeact-GFP to observe both SRM domains and actin patches. *cps1-191* is a thermosensitive mutant allele of *bgs1*$^{+}$. Cells were cultured in YES medium at permissive 25ºC (left panels) and restrictive 34ºC for 4 h (right panels), and imaged for filipin and GFP fluorescences.

(E) Time-course fluorescence micrographs of filipin-stained P*nmt1*$^{+}$-81-*bgs1*$^{+}$ cells carrying the non-functional correctly localized *bgs1*$^{9A(1136\text{-}1144)}$ mutant version and *crn1*$^{+}$*-GFP* to observe both SRM domains and actin patches in the absence of Bgs1 function but in the presence Bgs1 protein. Cells were grown at 28ºC in MM, transferred to MM + T, and imaged for filipin and GFP fluorescences using time-course microscopy at the indicated times with thiamine (0, 5, 10, and 15 h). Scale bars, 5 µm.

Figure S6.

Bgs1 localization is not dependent on either actin polymerization or SRM domains polarization.

Related to Figures 4, 5, and S5.

(A) Time-course fluorescence micrographs of filipin-stained cells carrying *RFP-bgs1*$^{+}$ in the absence of polymerized F-actin. Cells were cultured to early log-phase at 28ºC in YES medium (control). Latrunculin A (LatA, 100 µM) was then added, and the cells were imaged for filipin and RFP fluorescences using time-course microscopy at the indicated times with LatA (0, 30 min, 1, 2, and 3 h).

(B) Time-course fluorescence micrographs of *RFP-bgs1*$^{+}$ *crn1*$^{+}$*-GFP* or *GFP-bgs4*$^{+}$ cells grown in the presence of filipin. Cells were cultured to early log-phase at 28ºC in YES medium (control), then filipin (5 µg/ml) was added, and the cells were imaged for filipin, RFP, and GFP fluorescences using time-course microscopy at the specified times with filipin (0, 5, 15, 30, 45 min, and 1 h). After long times with filipin (60 min), there is a slight decrease in number (depolymerization), but not delocalization, of actin patches. Scale bars, 5 µm.

Figure S7.

Wsp1, Arp3, and Myo1 cooperate with the Tea1-Tea4 complex, but not with Bgs1, in the control of growth polarity.

Related to Figure 5.

(A) Phase contrast micrographs illustrating the rod-shaped morphology of *wsp1*Δ and the rounded morphology of *tea1*Δ *wsp1*Δ cells grown at 36ºC for 24h (upper panels). Similar dual morphologies are observed for *arp3-C1* and *tea1*Δ *arp3-C1* cells grown at 36ºC for 48h (middle panels), and for *myo1*Δ and *tea1*Δ *myo1*Δ cells grown at 36ºC for 24h (lower panels). Cells were cultured to early log-phase in YES medium at 25ºC, shifted to 36ºC to increase their putative deletion phenotypes, and imaged at the indicated times (24 and 48 h).

(B) Phase contrast micrographs depicting the typical Bgs1-absence morphology of *wsp1*Δ and *arp3-C1* cells carrying P*nmt1*$^{+}$-81-*bgs1*$^{+}$. Cells were grown to early log-phase at 28ºC in MM + S, transferred to MM + T + S, and imaged at the indicated times with thiamine (0 and 36 h). Scale bars, 5 µm.

Figure S8.

Bgs1 is required for endocytosis and membrane traffic from the plasma membrane to vacuoles.

Related to Figures 6 and 7.

(A) Kinetics of FM4-64 internalization in WT (upper panels) and P*nmt1*$^{+}$-81-*bgs1*$^{+}$ (lower panels) cells grown at 28 ºC in MM + T + S for 24 h (*bgs1*$^{+}$ OFF, +T), transferred to the same medium containing FM4-64, and imaged for FM4-64 fluorescence using time-course microscopy at the indicated times with FM4-64 (0, 3, 6, 9, 12, 15 and 18 min). Arrow, time of FM4-64 endocytosis in which most of the fluorescent dye is internalized (3 min in WT cells and 12 min in the absence of Bgs1).

(B) Fluorescence micrographs of FM4-64 internalization in P*nmt1*$^{+}$-81-*bgs1*$^{+}$ cells during *bgs1*$^{+}$ repression. Early log phase P*nmt1*$^{+}$-81-*bgs1*$^{+}$ cells were grown at 28ºC in MM, transferred to MM + T, and collected at the indicated times with thiamine. FM4-64 was then added for 60 min as in Figure 6B, and the cells were imaged for FM4-64 fluorescence using time-course microscopy at the indicated times with thiamine (0, 5, 10, 15, and 24 h).

(C) Differential interference contrast (DIC) and fluorescence micrographs of Blue CMAC-stained vacuoles

of P*nmt1*$^+$-81-*bgs1*$^+$ cells during *bgs1*$^+$ repression. Early log phase P*nmt1*$^+$-81-*bgs1*$^+$ cells grown at 28ºC in MM were transferred to MM + T and imaged for DIC and Blue CMAC fluorescence using time-course microscopy at the specified times with thiamine (0, 5, 10, 15, and 24 h). Scale bars, 5 µm.

(D) Scheme showing the timing of the emergence of phenotypes during *bgs1*$^+$ repression. After 10 h of repression, the cells started to exhibit simultaneously 1) dispersion of actin patches; 2) spread of SRM domains; 3) delayed endocytosis; and 4) membrane trafficking and fusion defects, giving rise to smaller numerous endosomes and vacuoles. After 15 h of repression, the cells started to display morphological defects of elongated and multiseptated cells in the presence, and spherical cells in the absence of the Tea1-Tea4 complex. It should be noted that during *bgs1*$^+$ repression cell growth progressively slows down and, therefore, after 10 h only intervals that cover a single cell cycle are shown (10, 15, 24, and 36 h).

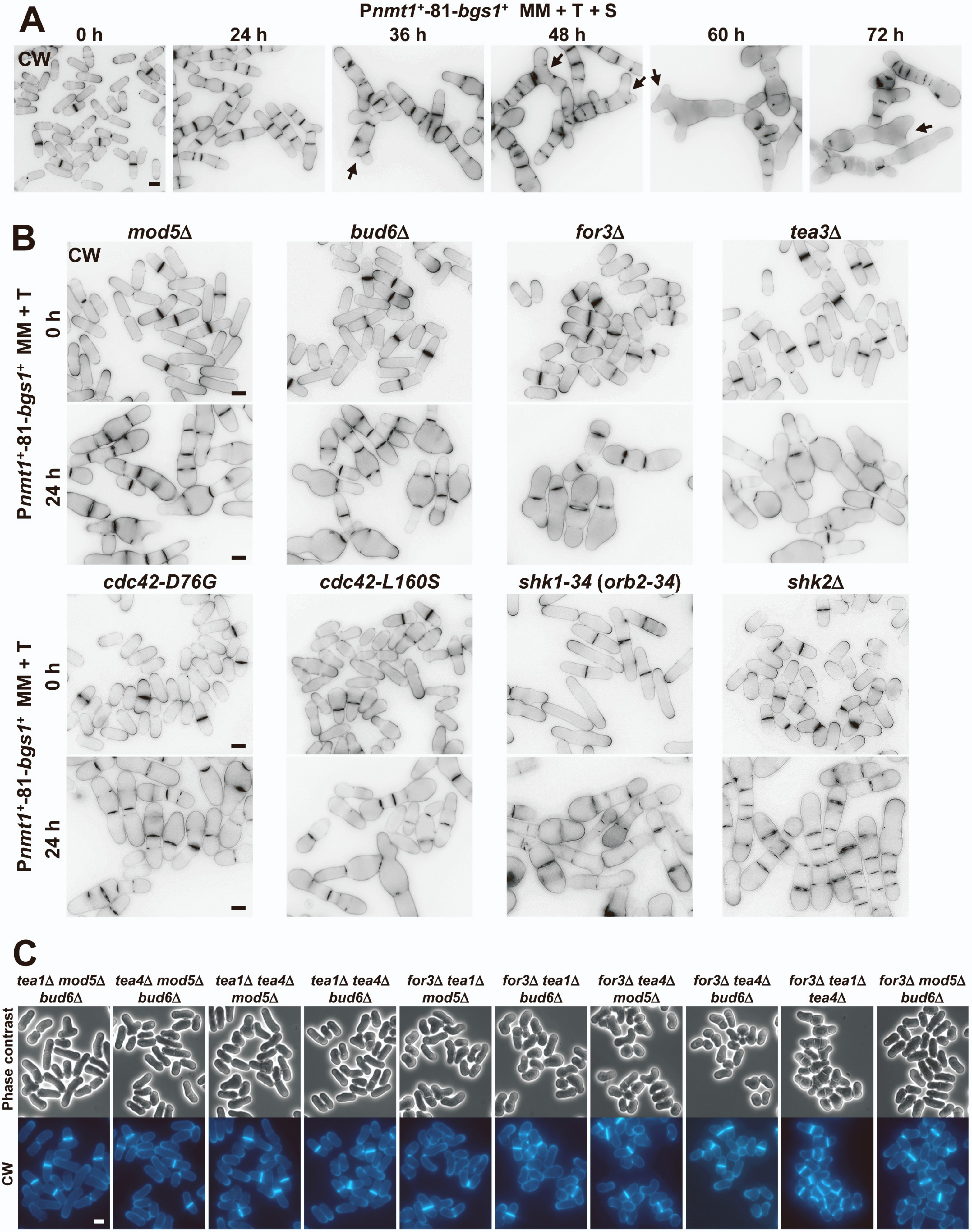
A
Pnmt1⁺-81-bgs1⁺ MM + T + S
0 h
24 h
36 h
48 h
60 h
72 h
CW
B
mod5Δ
bud6Δ
for3Δ
tea3Δ
CW
Pnmt1⁺-81-bgs1⁺ MM + T
0 h
24 h
cdc42-D76G
cdc42-L160S
shk1-34 (orb2-34)
shk2Δ
Pnmt1⁺-81-bgs1⁺ MM + T
0 h
24 h
C
tea1Δ mod5Δ bud6Δ
tea4Δ mod5Δ bud6Δ
tea1Δ tea4Δ mod5Δ
tea1Δ tea4Δ bud6Δ
for3Δ tea1Δ mod5Δ
for3Δ tea1Δ bud6Δ
for3Δ tea4Δ mod5Δ
for3Δ tea4Δ bud6Δ
for3Δ tea1Δ tea4Δ
for3Δ mod5Δ bud6Δ
Phase contrast
CW

Figure S1

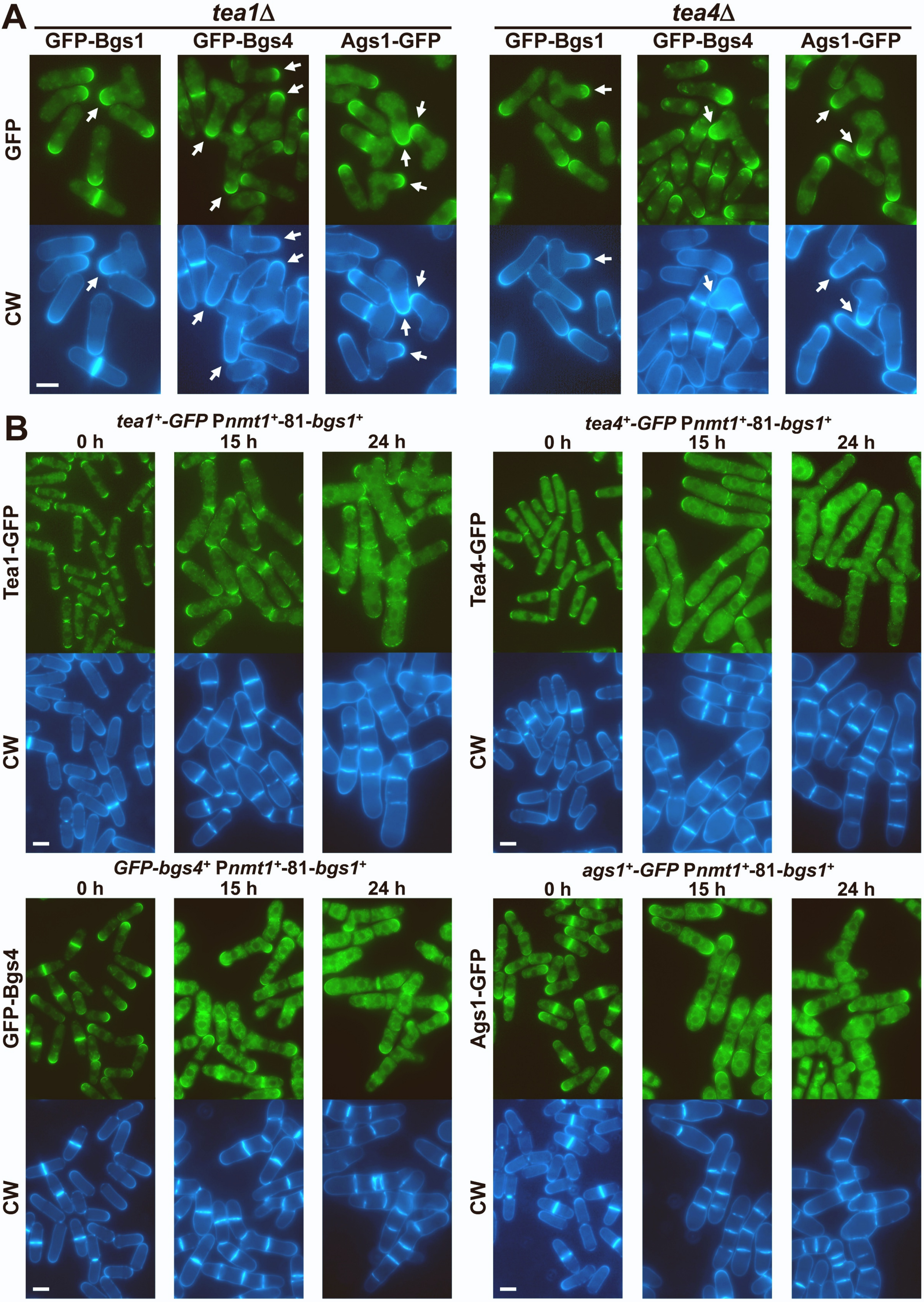
A
tea1Δ
GFP-Bgs1
GFP-Bgs4
Ags1-GFP
tea4Δ
GFP-Bgs1
GFP-Bgs4
Ags1-GFP
GFP
CW
B
tea1+-GFP Pnmt1+-81-bgs1+
0 h
15 h
24 h
Tea1-GFP
CW
tea4+-GFP Pnmt1+-81-bgs1+
0 h
15 h
24 h
Tea4-GFP
CW
GFP-bgs4+ Pnmt1+-81-bgs1+
0 h
15 h
24 h
GFP-Bgs4
CW
ags1+-GFP Pnmt1+-81-bgs1+
0 h
15 h
24 h
Ags1-GFP
CW

Figure S2

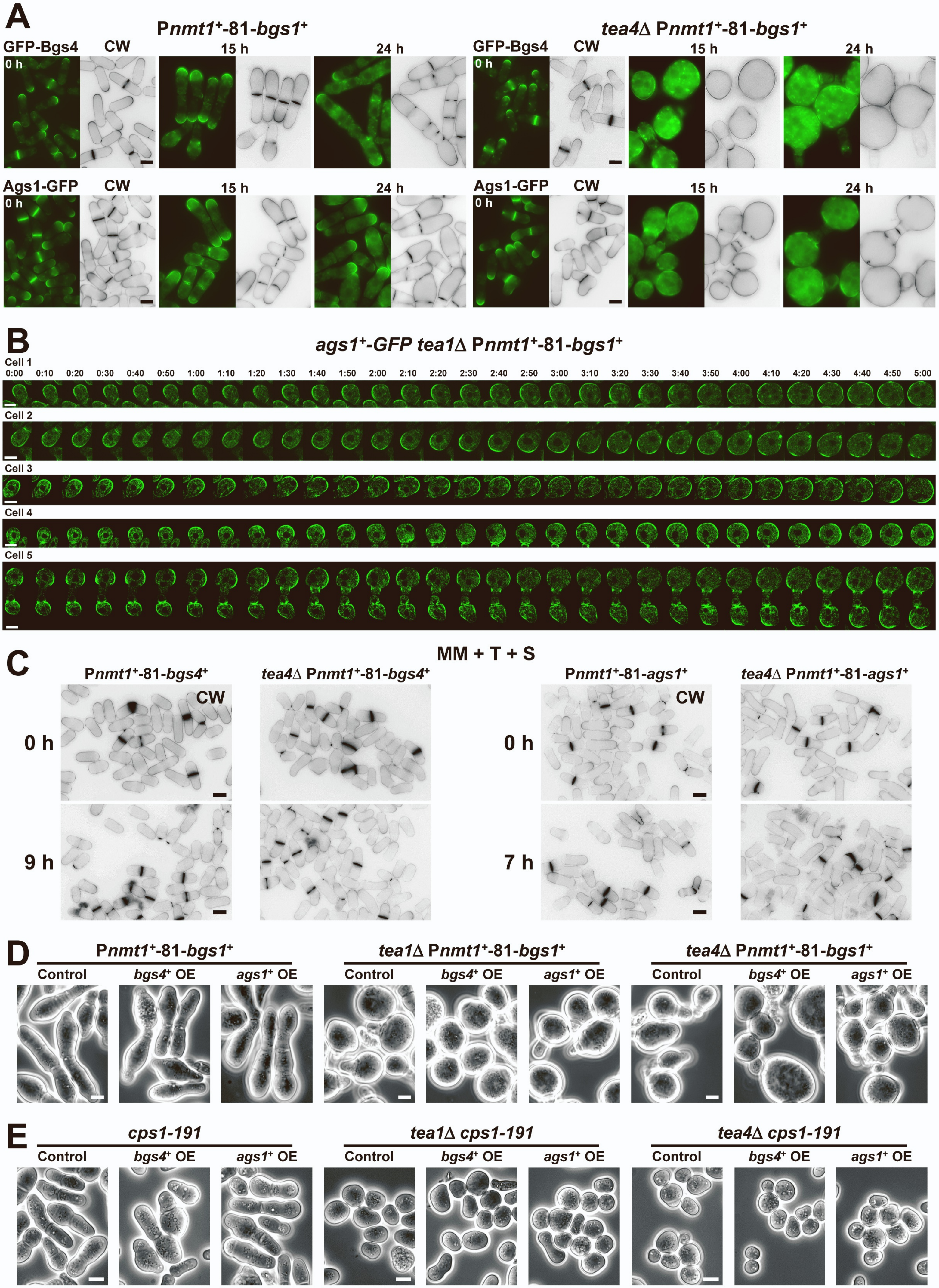
A
Pnmt1+-81-bgs1+
tea4Δ Pnmt1+-81-bgs1+
GFP-Bgs4
CW
0 h
15 h
24 h
Ags1-GFP
B
ags1+-GFP tea1Δ Pnmt1+-81-bgs1+
Cell 1
Cell 2
Cell 3
Cell 4
Cell 5
0:00 0:10 0:20 0:30 0:40 0:50 1:00 1:10 1:20 1:30 1:40 1:50 2:00 2:10 2:20 2:30 2:40 2:50 3:00 3:10 3:20 3:30 3:40 3:50 4:00 4:10 4:20 4:30 4:40 4:50 5:00
C
MM + T + S
Pnmt1+-81-bgs4+
tea4Δ Pnmt1+-81-bgs4+
Pnmt1+-81-ags1+
tea4Δ Pnmt1+-81-ags1+
9 h
7 h
D
Pnmt1+-81-bgs1+
tea1Δ Pnmt1+-81-bgs1+
tea4Δ Pnmt1+-81-bgs1+
Control
bgs4+ OE
ags1+ OE
E
cps1-191
tea1Δ cps1-191
tea4Δ cps1-191

Figure S3

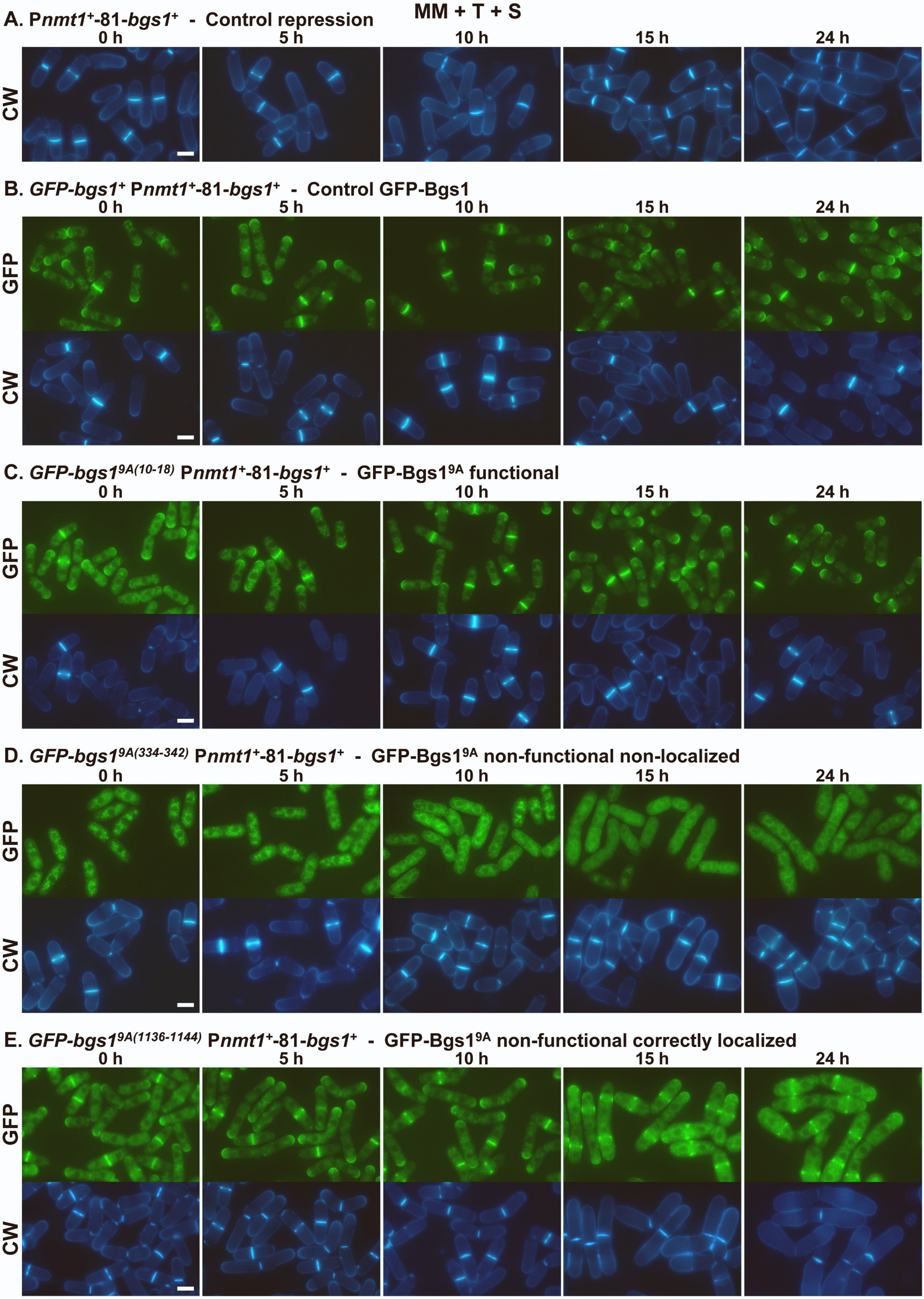
MM + T + S
A. Pnmt1+-81-bgs1+ - Control repression
B. GFP-bgs1+ Pnmt1+-81-bgs1+ - Control GFP-Bgs1
C. GFP-bgs19A(10-18) Pnmt1+-81-bgs1+ - GFP-Bgs19A functional
D. GFP-bgs19A(334-342) Pnmt1+-81-bgs1+ - GFP-Bgs19A non-functional non-localized
E. GFP-bgs19A(1136-1144) Pnmt1+-81-bgs1+ - GFP-Bgs19A non-functional correctly localized
0 h
5 h
10 h
15 h
24 h
GFP
CW

Figure S4

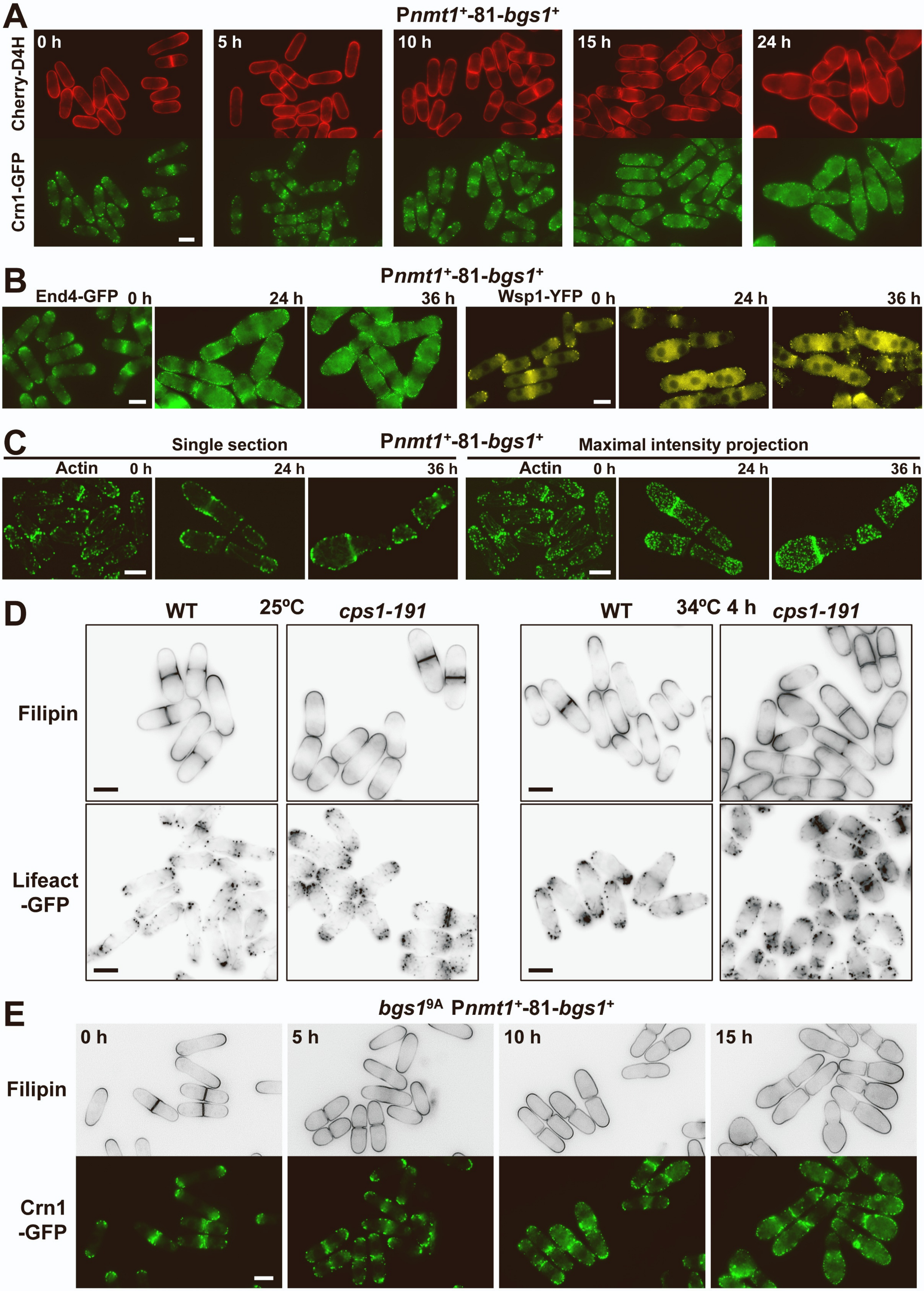

A
Pnmt1+-81-bgs1+
Cherry-D4H
Crn1-GFP
0 h
5 h
10 h
15 h
24 h
B
Pnmt1+-81-bgs1+
End4-GFP
0 h
24 h
36 h
Wsp1-YFP
C
Single section
Pnmt1+-81-bgs1+
Maximal intensity projection
Actin
D
WT
25ºC
cps1-191
34ºC 4 h
Filipin
Lifeact
-GFP
E
bgs19A Pnmt1+-81-bgs1+
Filipin
Crn1
-GFP

Figure S5

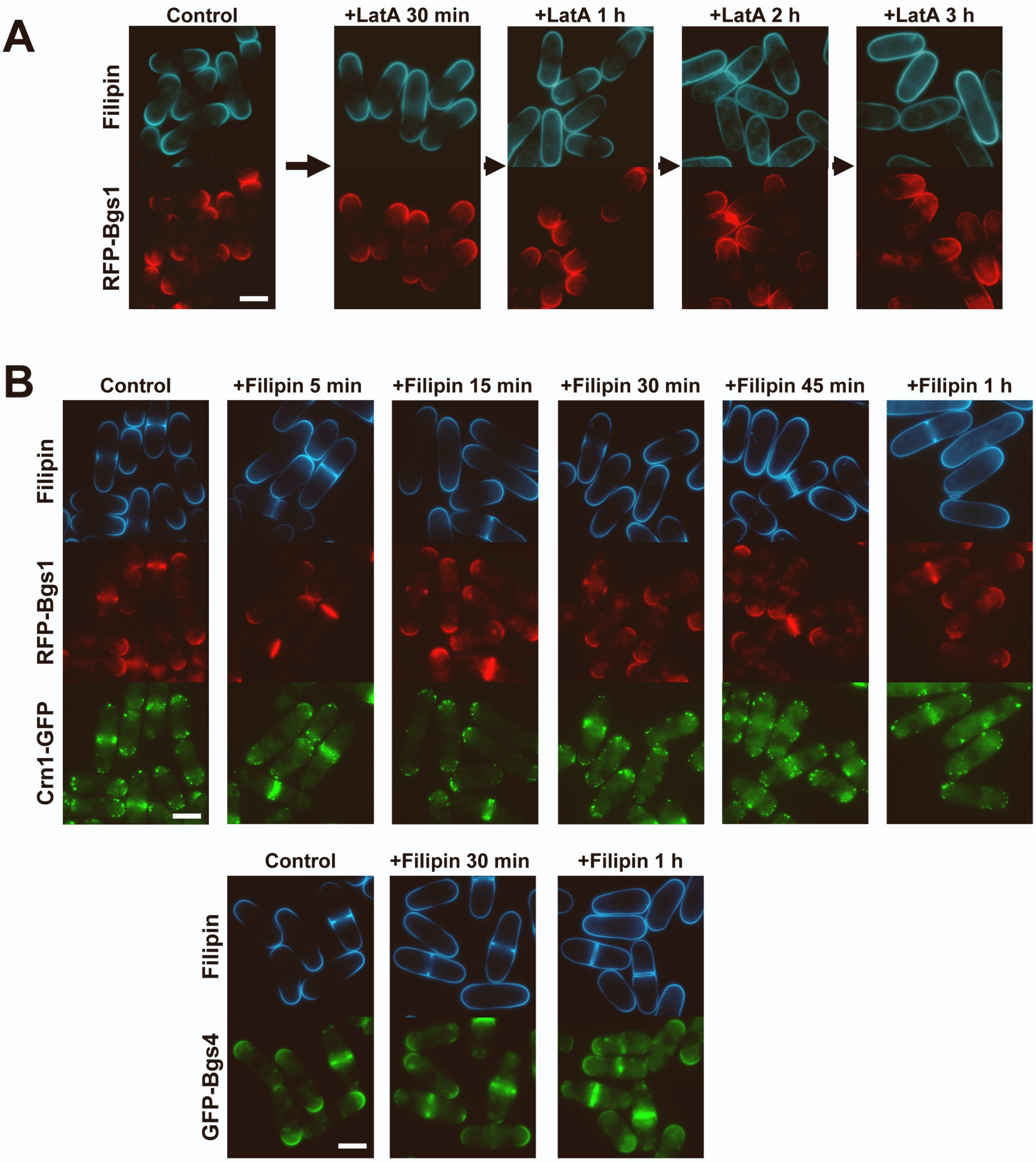
A
Control
+LatA 30 min
+LatA 1 h
+LatA 2 h
+LatA 3 h
Filipin
RFP-Bgs1
B
Control
+Filipin 5 min
+Filipin 15 min
+Filipin 30 min
+Filipin 45 min
+Filipin 1 h
Filipin
RFP-Bgs1
Crn1-GFP
Control
+Filipin 30 min
+Filipin 1 h
Filipin
GFP-Bgs4

Figure S6

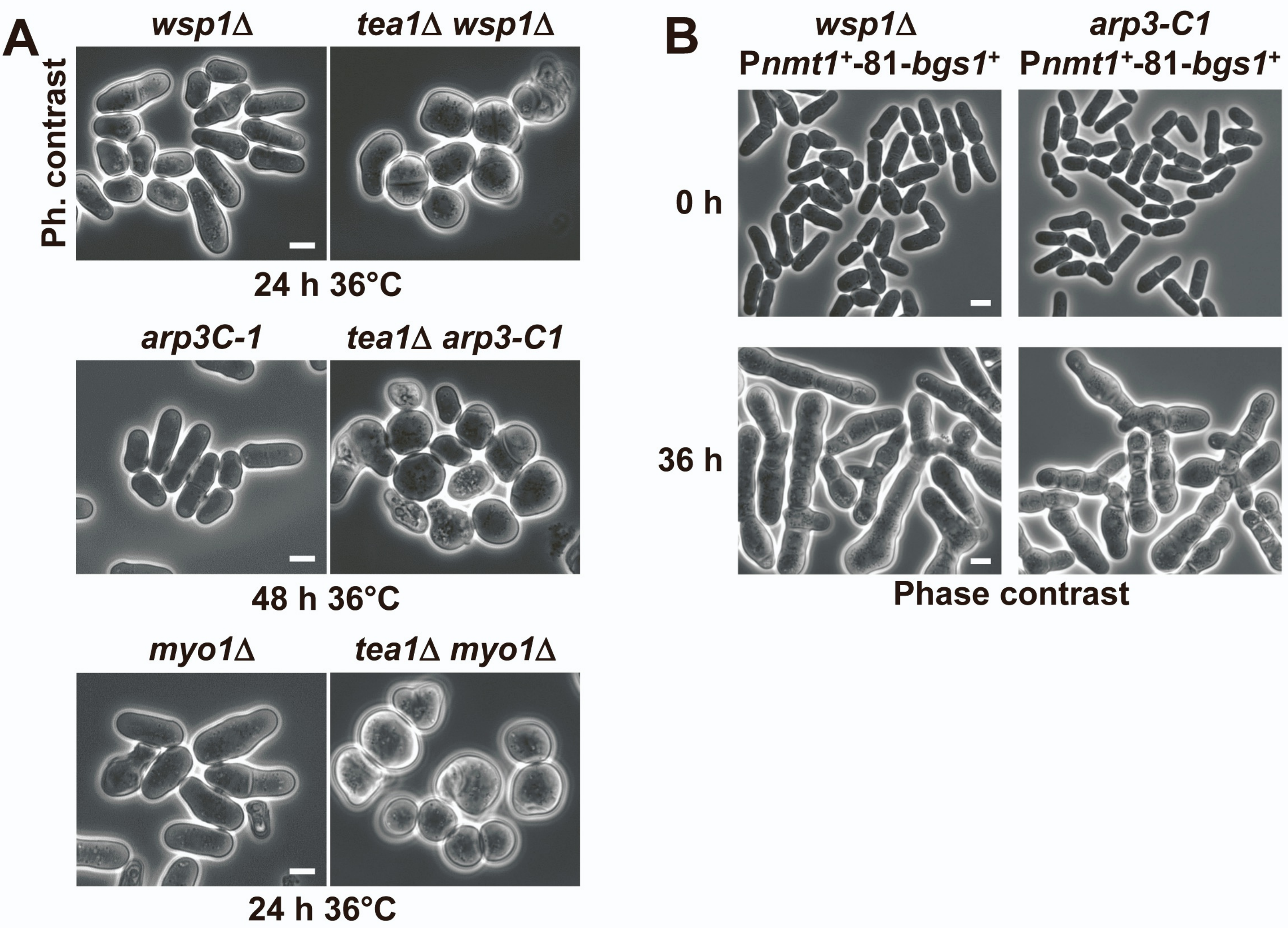
A
wsp1Δ
tea1Δ wsp1Δ
Ph. contrast
24 h 36°C
arp3C-1
tea1Δ arp3-C1
48 h 36°C
myo1Δ
tea1Δ myo1Δ
24 h 36°C
B
wsp1Δ Pnmt1+-81-bgs1+
arp3-C1 Pnmt1+-81-bgs1+
0 h
36 h
Phase contrast

Figure S7

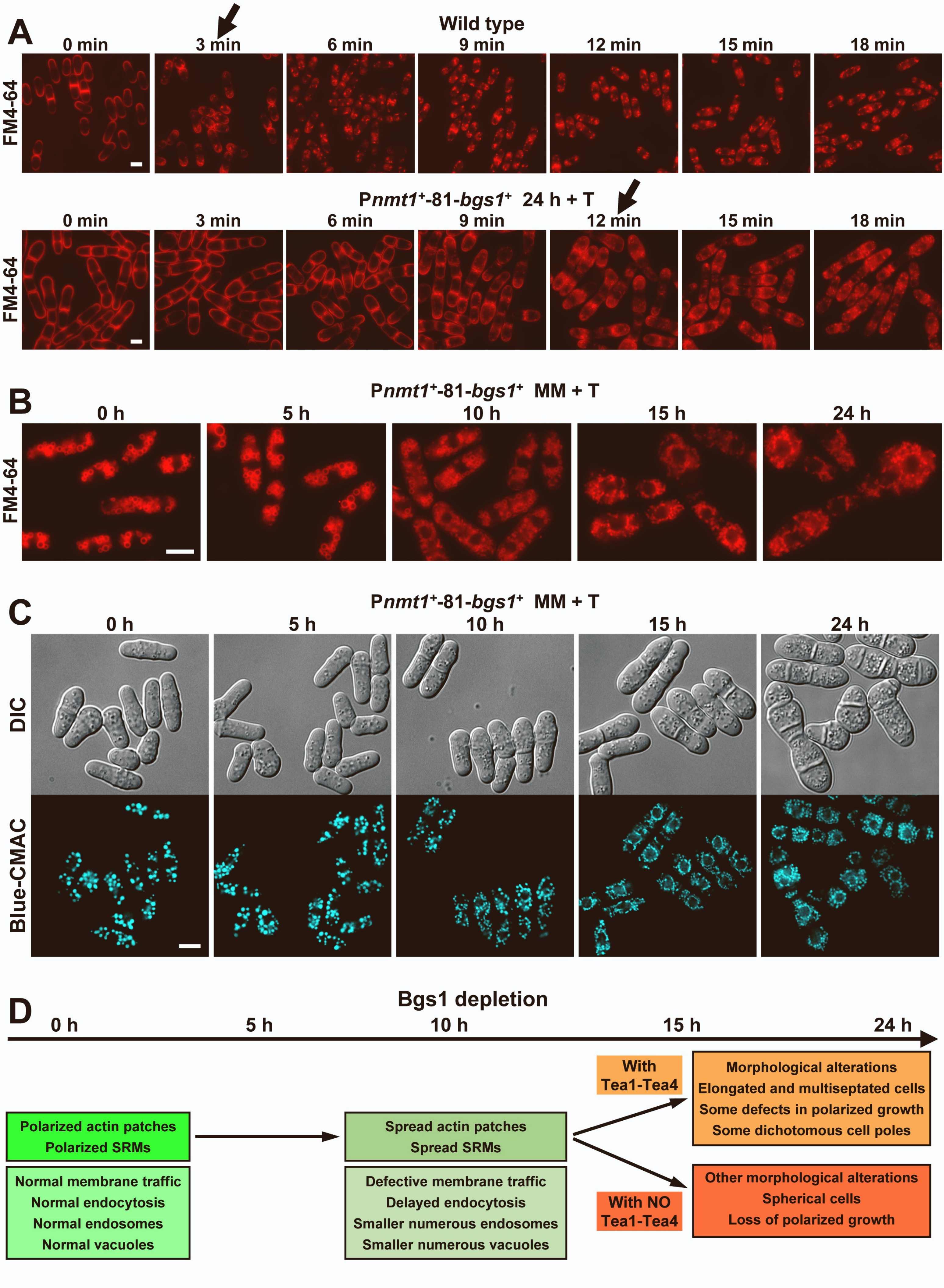
A
Wild type
0 min
3 min
6 min
9 min
12 min
15 min
18 min
FM4-64
Pnmt1+-81-bgs1+ 24 h + T
B
Pnmt1+-81-bgs1+ MM + T
0 h
5 h
10 h
15 h
24 h
C
DIC
Blue-CMAC
D
Bgs1 depletion
Polarized actin patches
Polarized SRMs
Normal membrane traffic
Normal endocytosis
Normal endosomes
Normal vacuoles
Spread actin patches
Spread SRMs
Defective membrane traffic
Delayed endocytosis
Smaller numerous endosomes
Smaller numerous vacuoles
With Tea1-Tea4
Morphological alterations
Elongated and multiseptated cells
Some defects in polarized growth
Some dichotomous cell poles
With NO Tea1-Tea4
Other morphological alterations
Spherical cells
Loss of polarized growth

Figure S8

TABLE S1. Summary of the morphological phenotypes exhibited by different combinations of multiple deletions of genes involved in cell polarity.

Related to Figures 1 and S1, and Table 1.

Strain	Cell morphology
*tea1*Δ *mod5*Δ *bud6*Δ	*tea*Δ phenotype (non-rounded)
*tea4*Δ *mod5*Δ *bud6*Δ	*tea*Δ phenotype (non-rounded)
*tea1*Δ *tea4*Δ *mod5*Δ	*tea*Δ phenotype (non-rounded)
*tea1*Δ *tea4*Δ *bud6*Δ	*tea*Δ phenotype (non-rounded)
*for3*Δ *tea1*Δ *mod5*Δ	*for3*Δ phenotype (non-rounded)
*for3*Δ *tea1*Δ *bud6*Δ	*for3*Δ phenotype (non-rounded)
*for3*Δ *tea4*Δ *mod5*Δ	*for3*Δ phenotype (non-rounded)
*for3*Δ *tea4*Δ *bud6*Δ	*for3*Δ phenotype (non-rounded)
*for3*Δ *tea1*Δ *tea4*Δ	*for3*Δ phenotype (non-rounded)
*for3*Δ *mod5*Δ *bud6*Δ	*for3*Δ phenotype (non-rounded)
